# An Encounter With the Other: A Thematic and Content Analysis of DMT Experiences From a Naturalistic Field Study

**DOI:** 10.3389/fpsyg.2021.720717

**Published:** 2021-12-16

**Authors:** Pascal Michael, David Luke, Oliver Robinson

**Affiliations:** ^1^School of Human Sciences, Old Royal Naval College, University of Greenwich, London, United Kingdom; ^2^Department of Brain Sciences, Faculty of Medicine, Centre for Psychedelic Research, Imperial College London, London, United Kingdom

**Keywords:** dimethyltryptamine, thematic analysis, naturalistic, field study, social, psychedelic

## Abstract

**Introduction:** *N,N-dimethyltryptamine* (DMT) is an endogenous serotonergic psychedelic capable of producing radical shifts in conscious experience. Increasing trends in its use, as well as new trials administering DMT to patients, indicate the growing importance of a thorough elucidation of the qualitative content, over and above structure, which the drug occasions. This is particularly in light of the hyper-real, otherworldly, and often ontologically challenging yet potentially transformative, nature of the experience, not least encounters with apparently non-self social agents. Laboratory studies have been limited by clinical setting and lacking qualitative analyses of experiential content, while online surveys’ limitations lie in retrospective design, uncontrolled use, and both of which not guaranteeing ‘breakthrough’ experiences, i.e., producing very strong psychoactive effects.

**Methods:** We report on the first naturalistic field study of DMT use including its qualitative analysis. Screened, healthy, anonymised and experienced DMT users were observed during their non-clinical use of the drug at home (40–75 mg inhaled). In-depth semi-structured interviews (inspired by the micro-phenomenological technique) were employed immediately after their experience. This paper reports on the thematic analysis of one major domain of the breakthrough experiences elicited, the ‘other’. Thirty-six post-DMT experience interviews with mostly Caucasian (83%) males (eight female) of average 37 years were predominantly inductively coded.

**Results:** Invariably, profound and highly intense experiences occurred. The first overarching category comprised the encounter with other ‘beings’ (94% of reports), encompassing super-ordinate themes including the entities’ role, appearance, demeanour, communication and interaction; while the second overarching category comprised experiences of emerging into other ‘worlds’ (100% of reports), encompassing super-ordinate themes of the scene, the contents and quality of the immersive spaces. Many further mid-level themes and subthemes also illuminate the rich content of the DMT experience.

**Discussion:** The present study provides a systematic and in-depth analysis of the nuanced content of the otherworldly encounter within the breakthrough DMT experience, as well as elaborating on the resonances both with previous DMT studies focusing on entity encounters and other types of extraordinary experiences entailing such encounters. These include the alien abduction, folkloric, shamanic and near-death experience. Putative neural mechanisms of these features of the DMT experience and its promise as a psychotherapeutic agent are discussed in light of such findings.

## Introduction

### DMT: ‘The Spirit Molecule’

*N,N-dimethyltryptamine* (DMT) is an indole alkaloid and potent serotonergic psychedelic, acting on the 5HT-2A receptor, which is virtually ubiquitous in nature (McKenna, 2018) and endogenous to humans. Reviews of its detection in the urine, cerebrospinal fluid and blood of human beings points to a maximum possible endogenous concentration of 55 ng/ml (Barker et al., 2012). Dean et al. (2019) have evidenced the presence of indole-ethylamine-methyltransferase (INMT), a key requisite enzyme for DMT synthesis, in the human cerebral frontal cortex and pineal gland.

DMT is one of the major active constituent in the Amerindian shamanic decoction, ayahuasca (‘vine of the dead’), typically blending the DMT-concentrated *Psychotria viridis* and *Banesteriopsos caapi* that contains the beta-carbolines rendering DMT orally active. DMT ingestion amongst humans has a potentially ancient history; it was identified, alongside harmine (one of the other ayahuasca beta-carbolines), in a ritual bundle from the Bolivian Andes dated to at least 1,000 CE (Miller et al., 2019). Use of DMT (and similar compounds; 5MeO-DMT and 5MeO-DIPT) in contemporary society, at least in the United States general population, is relatively rare at 0.7% (Palamar and Le, 2018). Research slightly prior to this finding suggest that its use may be increasing, as a larger proportion (24%) of DMT users were identified as new users compared with other psychedelics (Winstock et al., 2014). With its physiological role yet to be clarified (Rodrigues et al., 2019), given its profound psychoactivity as well as proclivity to engender subjective experiences of sensed presence, or interactions with other-than-self social agents, it has been suggested that DMT models or may be implicated in a host of altered states ranging from dreaming or psychosis (Dean, 2018), to spontaneous transpersonal experiences such as alien abduction experiences (Luke, 2012) – or near-death experiences (NDEs; Strassman, 2001; Timmermann et al., 2018), DMT’s precise qualitative relationship with which will be the dedicated subject of a subsequent paper.

Not many studies have been dedicated to systematic qualitative analyses of the psychedelic experience more generally (e.g., ayahuasca: Kjellgren et al., 2009; Wolff et al., 2019; psilocybin: Turton et al., 2014; ibogaine: Schenberg et al., 2017b; see below re: DMT), while some have explored the subjective mediation of psychedelic therapy (e.g., ayahuasca: Loizaga-Velder and Verres, 2014; Dos Santos et al., 2018; psilocybin: Belser et al., 2017; Watts et al., 2017; ibogaine: Schenberg, et al., 2017a; LSD: Gasser et al., 2015). Beginning with a surveying of DMT use in modern and indigenous Australia (Tramacchi, 2006), there has been a recent surge in interest in the phenomenology of DMT, including its cultural history, and the gnostic insight and essential liminality that is defining of the experience (St John, 2015, 2018; John, 2018). The remarkable consistency in many of its motifs has been noted (Gallimore and Luke, 2015) including the nature of the encounters with entities, which is itself a subtopic of significant attention, with direct implications for mechanisms of social cognition, and possibly for ontology (Luke, 2008, 2011; Luke and Spowers, 2018).

### Varieties of DMT Experience

In April 1956, Stephen Szara self-administered DMT intramuscularly (I.M.) – representing the very first human experience with pure DMT – and at 1 mg/kg reported sensations such as ‘brilliantly coloured oriental motifs and wonderful scenes altering rapidly’ (Szára, 2014). The study of Sai-Halasz et al. (1958) constitutes the very first research study in human participants, and used 0.7–1 mg/kg I.M. DMT in 30 healthy individuals. The report divided the experience into coarse and pathological categories, as symptomatic of such early DMT studies, including with patient populations (Boszormenyi and Szara, 1958; Turner and Merlis, 1959) – saliently however, incorporating ‘anxiety’ qualified with the statement, ‘when the world changes so suddenly and intensely that the subject loses his solid environment’ (Sai-Halasz et al., 1958, p. 9). Strassman (2001), in one of the first landmark psychedelic studies post-prohibition, documented the experiences of 60 participants undergoing over 400 DMT doses, which resulted in at least half of the *high-dose* (21–28 mg/kg, I.V.) participants reporting otherworldy adventures with other beings (Luke, 2013) – a closer content analysis of which will be the focus of a subsequent paper.

Constituting the only other thematic analysis of the DMT experience, Cott and Rock (2008) coded 19 narrative reports from an online survey, with a sample of mean age 23 (*SD* = 6), 95% male, and time since last DMT usage ranging from 2 days (11%) to 2 years (5%). Twenty percent were previously DMT-naïve (5% having used DMT >100 times). Nine very generic themes were identified, as follows – *Hallucinations*: which were visual, bodily, or auditory; *Veridical hallucinations*: entailing transportation to another world and supplanting of ordinary experience by ‘DMT hyperspace’, persistently considered to be true or real, and contact with sentient beings attributed ‘exosomatic ontological status’; *Affective distortion*; *Distortion in time, space and self*; *Spirituality*: denoting descriptions of beauty, love, religious experiences, or insights into self or the nature of the universe; *Familiarity*: denoting a sense of having been in the DMT space before (or even always residing there); *Lucidity*; *Ineffability*; and *Extreme intensity*.

Most recently, Timmermann et al. (2019), in the latest laboratory research with DMT, have provided a unique, valuable neurophenomenological study of the DMT experience, in which they illustrate intimate correlations between the phenomenological structure of first-person reports, specifically the ‘visual’, ‘bodily’ and ‘metacognitive/emotional’ domains they identified, and real-time neurophysiological measures. Lyke (2019) has conducted a content analysis of entity descriptions specifically, across 149 online published DMT trip-reports (90% male, mean age 25 years) from 2009 to 2019, which entailed 180 entity encounters in total. Comprehensively, Davis et al., 2020b undertook an international survey encompassing 2,561 respondents reporting ‘encounter’ experiences after a ‘breakthrough’ dose of inhaled DMT (that is, ‘one that produced very strong psychoactive effects’). Seventy-seven percent of the sample were male, slightly lower than the prior studies, though 85% were Caucasian/white, 64% living in the United States, and an average age of 32 (*SD* = 9). Respondents had used DMT an average of 14.5 times, and 67% reported that their most memorable entity encounter, about which they are answering the survey, was their first encounter.

Other schematisations include Meyer (1992), which incorporated a ‘hyperspace’ populated with intelligent, communicative entities in 66.5% of 340 online trip-reports (also see Strassman et al., 2008). Lastly, Shanon (2002) anthropological charting of the phenomenology of ayahuasca (whose principal component is DMT) culminates in the transportation ‘to another realm of existence, one which he or she feels to be very real’, where also taxonomised are the ‘supernatural beings’ observable in the ayahuasca world. Specific cross-referencing between the results of these particular studies and the current analysis’ themes is given thorough attention in the discussion section below.

### Rationale for the Current Study

The thematic analysis by Cott and Rock (2008) and the encounter survey of Davis et al., 2020b are significantly limited in the following ways: under-representation of the general population, respondents’ self-selection – but primarily, small sample size (only the former case), relying on, sometimes triaged, narratives (instead of interview) or fixed-answers, retrospective reports resulting in potential memory bias (deterioration or elaboration), phenomenological (versus qualitative) analyses, inability of monitoring or regulation of not only dosage but set and setting and potential contamination of the experience by use of other substances. The present naturalistic field study of DMT use aimed to address almost all of these limitations, by performing a large-sample, thematic analysis (including content analysis) of a discrete dataset of breakthrough DMT experiences with convenience sampled participants, extracting rich content by *in situ* semi-structured interviews (SSI) immediately post-experience. Also, contrasting with prior laboratory studies, Strassman (2001) reports were based on unsystematic bedside notes after doses (albeit with a large sample size) ranging from 7 to 28 mg I.V. DMT, and Timmermann et al. (2018) purely psychometric analyses (*n* = 13) resulted from 7 to 20 mg I.V. DMT with only a subset receiving high-dose – whereas this study aimed to analyse only high-dose breakthrough experiences. While the SSI’s of this study are consistent with methods in qualitative psychology, it also aimed to employ the ‘bracketing’ technique (a central function of micro-phenomenological interviewing; Petitmengin, 2006, 2017; Timmermann et al., 2019). This allows for a provision of inductive pre-reflexivity, that is, encouraging participants to avoid judgements of experiences into predefined cultural categories (such as ‘alien’, regarding DMT, which may otherwise lead to propagation of such memes) or confabulating by biased recourse to one’s conceptual repertoire, and to instead attentively describe the constitutive features in one’s own words.

The aim of the present paper is to achieve, *via* the above improvements, a finer and more naturalistic resolution, and thus greater understanding, of the DMT experience. Especially, the nuances of the qualitative content of the experience, over and above the more gross phenomenological structure (such as Timmermann et al., 2019; see Varela and Shear, 1999; Petitmengin, 2006, for this valuable distinction, e.g., the former being of a variable nature and the latter relating to more invariant processes). This is crucial given its increasing use amongst the population, and more importantly owing to the scheduling of the first clinical trials to administer this intense and fast-acting psychedelic to depressed patients (Steiner et al., 2020; D’Souza, 2021) as well as healthy participants (Liechti and Ley, 2020; Scheidegger, 2021). The paper’s particular focus lies in the ‘otherly’ phenomena evoked by DMT, of immersion into different environments which, crucially, gives way to engaging with non-self entities. This is later discussed in terms of similarity with other exceptional experiences, including possibly shared neurobiological mechanisms, all of which may have implications for therapy. While the psychedelic mystical experience has garnered most attraction as predicting clinical outcome (Haijen et al., 2018; Roseman et al., 2018), the profundity of the entity encounter itself has recently gained attention as to its therapeutic potential (Davis et al., 2020b; Lutkajtis, 2020). Other themes, spanning the particular experiences associated with the ‘self’ (vs. other) such as psychological and emotional effects, including experients’ personal and ontological interpretation and impact, i.e., phenomenological features especially pertinent to DMT’s therapeutic capacity, will be delineated in a subsequent paper.

## Materials and Methods

### Participants and Recruitment

**Figure 1:**
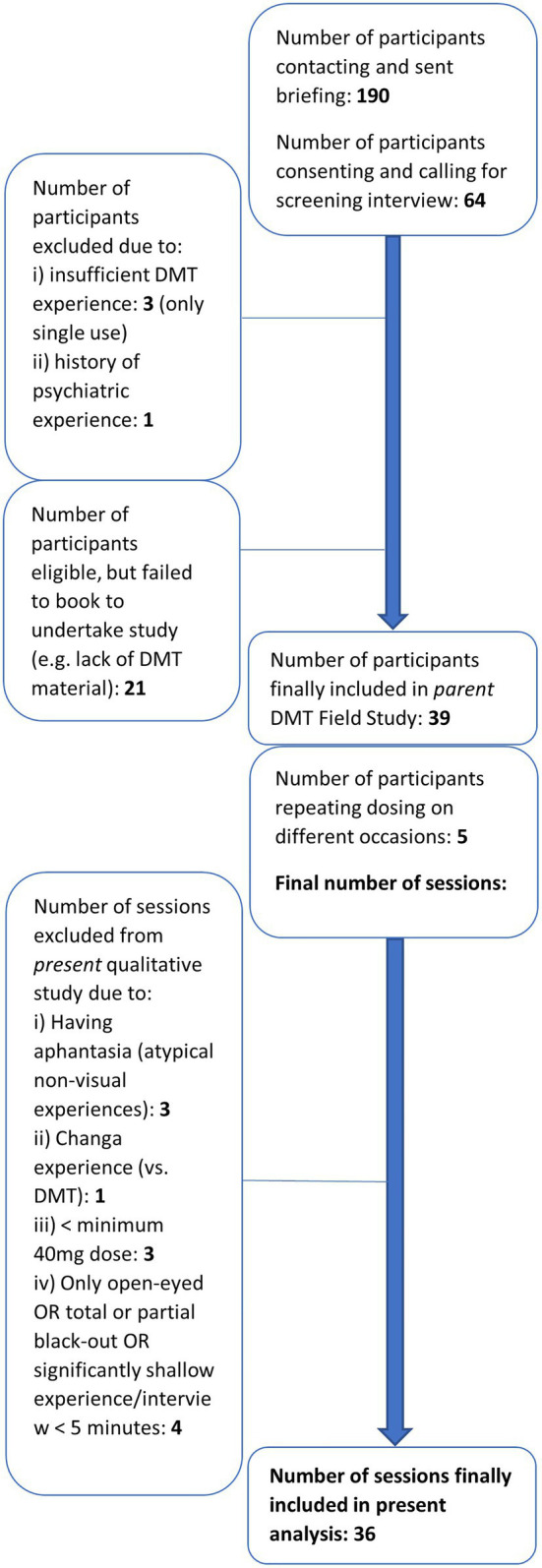
Participant recruitment flowchart – inclusion, exclusion, and final sample size overall and of present analysis.

Most volunteers were convenience sampled *via* online (social media) advertisement, with many sampled *via* snowballing. All prospective participants were subject to stringent inclusion–exclusion criteria. All recruited participants were experienced DMT users, with inclusion criteria entailing having taken DMT on three occasions, or at least significant other DMT analogous experiences such as ayahuasca or 4-AcO-DMT, with at least one ‘breakthrough’ experience (typically highly intense and perceptually immersive or remodelling; see Table 1). Participants were also required to provide their own DMT supply, exclusively of the form *N-N-dimethyltryptamine* (N-N-DMT; hereafter, referred to as DMT), and to live within the Greater London, Sussex or Kent area of the United Kingdom. Participants were selected in accordance with Johnson et al. (2008) volunteer safety guidelines for hallucinogen research, with exclusion criteria entailing prior psychedelic experiences which were difficult, i.e., putting self or others at risk, or leaving lasting psychological harm to self. The Structured Clinical Interview for DSM-IV Axis-I Disorders – Clinical Trials version (SCID-CT; First et al., 2007) was utilised by a trained SCID administrator as a screening tool, also excluding those with a current or past history of meeting DSM-IV criteria for schizophrenia or other psychotic disorders, or bipolar I or II disorder, as well as individuals with a first-degree relative with these disorders. Also excluded were those with lifetime presence of any major depressive or manic episode, substance abuse or dependence within the last 5 years, and any other current psychiatric diagnosis. Sixty-four prospective participants were screened, where 39 were finally included, resulting in 47 DMT sessions in the parent field study. Thirty-six sessions were the basis of the present thematic analysis, after removal of interviews not meeting several criteria, such as minimum use of 40 mg DMT (see Figure 1 for details).

**Table 1:** Participant demographics and DMT experience: average age, 37.4 (range: 23–58), eight female, 83% caucasian; AYA, ayahuasca; N/A, unprovided (free to withhold information due to illicit activity admission).

Participant number	Pseudonym	Age (range)	Sex	Nationality	First time DMT used	Last time DMT used	Overall times DMT used	% break-through DMT experiences (%)
1	MP	45–49	M	White British	2011	11/2016	20	33
2	TM (three doses)	30–34	M	White Romanian	2015	11/2016	5–6	100
3	BB	35–39	M	White British	2013	02/2018	15–20	25
5	JM	35–39	M	White British (Scottish)	2015	03/2017	12	66
6	RV	40–44	M	White British	2015	08/2018	1 (+4 AYA)	75
7	TC	25–29	M	White German	2014	06/2018	10–15	100
8	HV	35–39	F	Black British (Ghanaian-Egyptian descent)	2016	02/2018	80	<100
10	GR	25–29	M	White Romanian	2015	2015	2 (+4-ACO-DMT)	Once
11	SP (two doses)	35–39	M	White British	2003	06/2017	10–15	>50
12	RH (three doses)	55–59	M	Asian British (Indian descent)	2013	08/2018	Hundreds	75
14	AZ	25–29	M	Isreali	2013	02/2018	7	>40
15	ZD	30–34	F	White British	2017	03/2018	20	90
16	RS	25–29	M	Black British (African descent)	2016	05/2018	40	50
17	LR	25–29	M	Chinese-Italian (Dual)	2010	2011	25	40
23	AF	40–44	F	White Italian	2018	05/2019	2 (+8 AYA, 10 changa)	>75
24	LG	30–34	M	Mixed British (Sri Lankan-German descent)	2011	07/2019	20	20
25	AN	25–29	F	White British	2018	07/2019	7	>40
26	EM	20–24	F	White Romanian	2017	05/2019	10	90
27	AB	35–39	M	White British	N/A	N/A	10	100
30	SH	30–34	F	White British	2007	2008	6	50
32	OR (two doses)	25–29	M	Brazilian	2012	2018	3 (+Hundreds AYA)	Once
34	FF	45–49	M	White British	N/A	N/A	10	80
35	JB	40–44	M	White British	N/A	N/A	8	75
36	BW	45–49	M	White British	2000	07/2019	3 (+4 changa)	Once
40	JA	35–39	M	White British	2014	10/2019	70	>70
41	AV	45–49	F	Brazilian	2003	06/2019	20	100
42	MS	55–59	F	Mixed British (Iraqi-Italian descent)	2013	2017	5	100
43	DD	40–44	M	White British	N/A	N/A	Over hundred	>40
44	DS	45–49	M	White British	N/A	N/A	Hundred	50
47	ST	35–39	M	Nigerian	N/A	N/A	3	66

### Measures and Materials

The DMT material was weighed using 0.001 g microscales to monitor dosage. Given their experienced nature, ‘breakthrough’ doses were invariably desired by participants leading to a minimum dose of 40 mg (max. 75), and mean of 54.5 mg (*SD* 9.8; a crude approximate equivalent of at least 18 mg I.V.* to loss by exhalation, bioavailability and pyrolysis), inhaled *via* participants’ own smoking paraphernalia (almost always glass pipe. RH: Vape pen; DS and DD: ‘Dab rig’).

A detailed semi-structured interview was employed to explore the resultant DMT experience (see Supplementary Material 5). After recounting the experience as entirely and chronologically as possible, interview probes for elaboration spanned domains including entity encounters and visionary environments, as well as sensorial, bodily, emotional and cognitive experiences, corresponding (except the former two) to some of the major categories of the Hallucinogen Rating Scale (HRS), which was employed in the original studies of DMT administration (Strassman et al., 1994).

### Procedure and Anonymity

The field study and present analysis was approved by University of Greenwich Research Ethics Committee (Ref. 17.3.5.15). Given the class-A legal classification of DMT as a controlled substance, strict anonymity of all participants was held throughout. After reading of the online briefing, participants were given the option to consent (repeated again upon screening call and study day) and followed a link to book a timeslot to call a provided phone-number for the SCID-CT screening interview and were advised to block their caller ID. During the call they were asked to provide their chosen pseudonym by which they were to be called for the study’s duration. If they were eligible after screening, a mutual available time between subject and researcher was agreed to book when they plan to take the DMT, while establishing a meeting place from which to lead experimenters to their home or other suggested location agreed by researchers, to serve as the setting for the experience and study. In this way, no personal, identifiable information from the participant such as name, phone number or address was shared or recorded, and all reporting of data was anonymised. Naturalistic field research with psychoactive substances has previously been successful with ayahuasca and with neural and psychometric measures of DMT (Kuypers et al., 2016; Pallavicini et al., 2020).

Intensity ratings were asked of each participant (who stated their willingness to be asked) at 1 min time-intervals during their DMT experience, on a scale of 1–10 (1 = baseline; 10 = most intense experience ever experienced). The SSI was conducted immediately after the subsiding of the DMT experience, as indicated by participants’ indication of intensity being 1/10, and most often lasted at least 30 min (approximate range 12–75 min).

### Analyses

Interviews were audio-recorded by the researchers and deleted once uploaded to a secure cloud-storage system. All 45 (of 47, as two resulted in no experience) interviews were transcribed *verbatim*, including each of the 36 interviews reported herein. These were then coded with software package *NVivo* v. 12 (Windows).

The interviews were subjected to thematic analysis using a hybrid deductive-inductive process, wherein many of the eventual highest-order, overarching *categories* were based on, but not identical to, most of the major categories dividing the HRS (including ‘perception’, ‘somaesthesia’, ‘affect’, ‘cognition’; Strassman et al., 1994). After this point, all analyses and coding efforts were in accordance with the guidelines as provided by Braun and Clarke (2006), as well as being purely inductive in nature with codes and latter themes elicited by the interview data only. Importantly for the present paper focusing on only a subsection of wider analyses (the ‘Other’), this deductive aspect is not applicable here given the two overarching categories (elaborated in Results below) not being included in the HRS, and as such all analyses herein are inductively developed.

The extensively iterative thematising process predominantly comprised thorough re-reading of the narratives, the noting of preliminary concepts as initial codes which were then clustered according to thematic similarity into provisional but usually highly specific lowest-order *subthemes*. Such subthemes were then subsumed under the pre-determined overarching categories, and the process was repeated for each of the 36 interviews. The process of reviewing these many subtle themes, collated within the few broad categories, only until all interviews were coded allowed for more resolved identification of distinct or comparable concepts across the entire scope of interviews. This then led to construction of *super-ordinate* and even finer *mid-level themes*, both subsumed by the overarching categories but subsuming the lowest subthemes. Further refining processes included merging of redundant themes captured by other themes, or expansion of themes covering disparate concepts. Final naming of the themes, especially super-ordinate and mid-level, was decided upon once all subthemes were finalised to ensure a thorough capturing of total explored content.

When using the software *Nvivo*, the number of interviews (as separate documents) ascribed to each given code is automatically calculated. The frequency across the whole dataset with which each theme occurs is thus recorded, and this both serves to provide a content analysis, as well as highlighting the repeated nature of many highly specific themes across reports. Finally, during the write-up, an idiographic emphasis was assumed by reporting many participants’ accounts to extensive degrees, to illuminate the details of individual subthemes’ content over and above the superficial, summated, and thus nomothetic, overarching categories.

## Results

The following table (Table 2) presents all levels of themes to be described in the present paper’s qualitative analysis of the DMT experience – the two overarching categories comprising ‘Other beings’, and ‘Other worlds’. This is except all final subthemes, which are listed in Supplementary Table 3 in the Supplementary Material, where extra clarificatory notes for many of the themes can be found. Also available in the SM is a graphical representation of the themes (Supplementary Material 4). Both in the tables and the ensuing descriptions of themes, **bold** signifies super-ordinate themes (e.g., **Role** of the beings); *Italics* signify mid-level themes (e.g., Beings as *Showing*). In the descriptions of themes only, final subthemes (e.g., Beings as ‘Presenter’) are flanked by ‘apostrophes’ (and number of participant interviews in which final subthemes are present, out of total interviews, are shown in parentheses).

**Table 2:** Thematic analysis of the DMT experience: an encounter with the other; tabularisation of categories, super-ordinate and mid-level themes explored in the present article – see Supplementary Table 3 for list of all subthemes.

An encounter with the other	No. interviews/36 (%)
*Encountering other beings*	
*Transformation of persons present*	7 (19)
*DMT personification*	5 (14)
Sensed presence	
*Presences (No imagery)*	6 (17)
*Omnipresence*	5 (14)
Role and function: entities fulfilling elaborate, inter-relational purposes	
*Helping or nurturing*	19 (53)
*Showing or communing*	17 (47)
*Manipulating or controlling*	6 (17)
Appearance and features: a myriad of entities manifesting	
*Human*	6 (17)
*Other animals*	4 (11)
*Otherly creatures – non-human/non-animal*	26 (72)
*Sentient structures*	9 (25)
*Specific features*	9 (25)
*Visual quality*	19 (53)
Demeanour and nature: entities as magnificent, mischievous or menacing	
*Charming and inviting*	20 (56)
*Other dispositions*	12 (33)
*Mischievous or jestful*	5 (14)
*Fearsome or menacing*	3 (8)
*Nature*	17 (47)
*Expectation of subject*	6 (17)
*Gender*	17 (47)
Communication and messages: entities as instruments of personal and universal insight	
*Communication mode*	14 (39)
*Messages received*	12 (33)
Interaction and behaviour: entities as interfacing or independent	
*Active involvement*	10 (28)
*Passive activity*	19 (53)
*Exploring other worlds*	
*Breaking through the veil*	8 (22)
*Emergence into novel reality*	21 (58)
*Navigation through space*	11 (31)
The scene: from natural to artificial worlds	
*Human worlds*	6 (17)
*Natural worlds*	10 (28)
*Artificial worlds*	6 (17)
*Children’s worlds*	3 (8)
*Nebulous worlds*	13 (36)
The contents: from organic-mechanic to abstract objects	
*Organic objects*	16 (44)
*Technological objects*	8 (22)
*Infantile objects*	3 (8)
*Geometric objects*	16 (44)
*Symbolic objects*	6 (17)
*Miscellaneous objects*	14 (39)
The quality: a mixture of textures and transformations	
*Transforming or exploding*	7 (19)
*Synthetic textures*	15 (42)
*Generic textures*	11 (31)

### Encountering Other Beings

Thirty-four of thirty-six experiences (94%) incorporated an encounter of some nature with sentient entities that were experienced as beyond the self. These were highly complex dimensions to the DMT experiences, all of which involving characteristics such as a definable disposition, mode of interaction and most (29, 81%) with individualised visual form. However, some participants did describe sensed presences. This may have been either discreet *Presences* with no imagery, as six participants conveyed, for instance JM reporting ‘other vague hints at characters…more areas which had personality’. Or as how five participants articulated, where the presence in fact felt more like an *Omnipresence*, for example, SH invoking her understanding of tantric philosophy, ‘the Shiva principle is the kind of indivisible consciousness that is everything, that is also the manifest universe that’s Shakti – so it kind of felt like that’. It is noteworthy that 4/5 of these last participants were of the total 7 who at no point reported a more defined encounter accompanied by a visual form and behaviour. Additionally, five participants had a tendency to refer to *Personification of the DMT* itself, such as ‘the DMT was showing me around… it felt very feminine…pulling me into these vortices’ (SH). Finally, three participants also experienced the *Transforming of those present* in the room in different ways, for example ‘you just melded in with this bright light and you became this angelic shaman kind of guy’ (LR).

#### Role and Function: Entities Fulfilling Elaborate, Inter-relational Purposes

Each case of interfacing with these beings was also classified in terms of a specific role the majority of them played, reflecting their function and purpose while actively engaging with the experient: *Showing or Communing, Helping or Nurturing*, and *Manipulating and Controlling*. TM (Trip 2) reported a particular encounter illustrative of the first role *Showing or Communing*, reported by 17 individuals and mainly comprising of beings acting as a ‘presenter’ (10; hereafter all numbers in parentheses are the number of participants out of 36 reporting this feature) usually of intriguing objects, and ‘focuser’ (5) of their attention upon them:

“A lot of very strange clowns…mechanical entities. *Very* cartoonistic. And again trying to show me something… It was like a toy …continuously moving and changing shapes and colors… [they would] push them in my face… and I could see every single detail”

TM continues to encompass the *Helping or Nurturing* function, the most often reported, by 19, i.e., over half the participants, entailing the most benign aspects such as serving as an ebullient ‘playmate’ (5) or reassuring ‘soother’ (5):

“Floating, jumping around, very happy to show me those things, [and] that I’m there, but again I could not play with them, that game that they asked me to do…I’m paying attention to them…this entity was asking me to look at his toy, then another ask[ed] me ‘No, look at my toy, play with me!’… Very childish in what they were doing… Yeah, those entities calmed me down…their message somehow…gave me [the] impression that ‘everything’s ok, I’m in [a] good place, I do not need to worry, I’m too scared for nothing, and let us play with these toys!’”

The role of *Manipulating and Controlling*, in the minority at six reports, was very clearly conveyed by DD’s encounter with a Faerie-like entity who embodied the multifaceted ‘trickster’ archetype (Jung, 1969) (3) – that is, integrating other themes, i.e., the demeanours of being mischievous and childish, as well as the activities of laughing and tempting – who was also an ‘orchestrator’ (2) of the DMT-space:

“A female face which was tempting me, which I’m kind of familiar with… Then, everything slipped, everything stripped away. As I was tempted in, lured in… and there was like a ‘Hehehe’, there was a mischievousness to it. She was definitely giggling, as if to say, ‘This fucking stupid idiot here he comes again I’m gonna show him, Bang!’… She’s beckoning with a sort of finger like this, going ‘come in, come in’, and I thought OK, I’m mesmerised, so I follow her in… This female entity is fucking all-powerful but at the same time very mischievous and jokey and infantile… So she’s like, ‘I’m the master and orchestrator of this whole situation, but I like having a laugh at the same time’… In that space it’s almost like she came to get me, kind of knew I was coming – ‘Come, come, come, giggle giggle, laugh, look at that, now you are here, now I just sit back and watch you!’”

He reveals at the end, perhaps paradoxically (apt to trickster territory, given the ambivalence and boundary-breaking of the archetype) a protective ‘guardian’ (4) manifestation of the *Helping* role as well:

“She wasn’t a god-like deity…she was like a child God!… Sometimes very nurturing and maternal, very protective if she feels like I’m getting into bad, deep water”

#### Appearance and Features: A Myriad of Entities Manifesting

The ways in which participants articulated the overall appearance of their perceived entities, and the defining features and aesthetic thereof, yielded the greatest number of subthemes – 48 in total, all of which listed, alongside all other mid-level and subthemes, in Supplementary Table 3.

*Human beings* were seen only in six cases, and only in two of these were they persons known to the subject – and of interest, both of whom were deceased. The great majority, 26 reports, composed of forms attributable in some way to some *Otherly Creature*, that is of a non-human and non-animal nature. These infrequently consisted of more simplistic ‘silhouettes or featureless’ figures (5) – but ‘humanoid’ was the most common (9) generic shape to these creatures, mirroring their human experiencers, albeit with the vast array of inhuman manifestations. We have already met TM’s (Trip 2) cartoonistic mechanical clowns, with three other ‘jester’ or harlequin-like entities also encountered, while some other entities assumed ‘insectoid proportions’ (4), such as one ‘multidimensional moth-like structure’ (MP).

However, TM’s first DMT experience perfectly portrays a very colourful number of the elements of the *Otherly Creature* variation, specifically fusing ‘octopoid’ (4) and ‘serpentine’ (3) features while incorporating elements of elongation, popularly associated with the ‘Grey- or Mantis-like’ aliens (2):

“If I need to compare with something on this planet…they are like octopus, from…another planet. They had many similarities, like that long head…the scalp here was big… It’s far from octopus, but that’s the closest… Long heads and faces… They did not have the nose I think…they had a flat face somehow, maybe they had some holes under that mask… and their eyes were much bigger than normal… Yeah, I wasn’t scared, it was like alien eye, [a] big one. No it was like an animal, lamb…feeling only kindness… Very long hands…

I could see very strange hair…very similar to very thin and long snakes, kind of snakes…long and moving by itself”

*Sentient structures* were observed in nine cases, objects normally considered inanimate were animated. The entoptic displays were sometimes considered ‘sentient geometries’ (3), described as ‘hyperdimensional…dancing lattices’ and ‘unbelievable hyperintelligent geometry’, while ‘boxes’ (1) topped with ‘transparent interlocking mobius strips going round and round’, and ‘computer symbols’ (2) at the onset of the trip’s ‘start screen’ were additionally experienced.

The *Visual quality*, that is the texture with which these presences were furnished was also very diverse. ‘Self-transforming’ (as per ‘machine elves’, McKenna, 1993), a tendency to morph in a dynamic state of flux, was the most oft-reported, alongside being ‘geometric’ in aesthetic (8). Sometimes the geometric backlight of the DMT space itself informed the entities’ forms as if emerging form it. Of the four insectoid encounters, two were explicitly described as such; MP’s moth was ‘made of the same geometry…but wire-frame’, which was also ‘hyperdimensional’ (3), and ST’s grasshopper-like familial beings were described as ‘if light was coming from behind the curtain, how you would see silhouettes… The flower of life overlaid over everything…I could see an entity coalescing’.

The experient EM conveys the ‘self-transforming’ (8) nature of her ‘construction with a mind of its own…moving around, everything was changing inside’, closely echoing RH’s (Trip 1) living structure, a ‘honeycombed…massive building…evolving and changing’. However, EM also qualifies hers by stating ‘it was like this massive machine that keeps everything together’, referring to a further quality of being ‘mechanical’ (3) – and while not articulated as a sentient edifice, JM’s writhing environment has resounding parallels: ‘lots of wheels, cog-like representations, things spinning in multiple layers… They would all speed up as part of a larger geometry…mechanical movement… fluid, like a wind you could see’ – whose self-transforming machine world was, incidentally, inhabited by entities of an ‘elf’-like mischievousness.

FF’s following encounter mimics EM and JM’s above, in being apparently contained within the fluctuating ‘innards of a machine’ (see *Artificial worlds*, **Scene**), but the populace therein had a peculiarly simultaneous ‘organic-mechanic’ (1) aspect to them:

“There is a recurring image of like a space invader figure, sort of like an icon… It was definitely…maybe your blood flowing through your system… These things…were the workings of something!… [They] were following along these sort of trackways which were doing something, part of a bigger, maybe it was keeping me or keeping something alive…like seeing the workings of possibly myself or the human form, being inside seeing it maybe from a microscopic perspective. But it was much more mechanical and sort of alien… definitely more spacey…

Interviewer: And that tiny scale might blur the boundaries of what looks organic or mechanical?

Yeah absolutely!… there’s definitely life in there, or some sort of sense of being… It was almost like a ‘marble run’…like roller coasters, loads of these track ways where these things were moving along them”

#### Demeanour and Nature: Entities as Magnificent, Mischievous or Menacing

As many of the excerpts already provided will attest to, the great majority of the entities with whom participants connected came in peace – their disposition cultivated a positive encounter, and sometimes overwhelmingly so. Over half of cases, 20, involved *Charming and Inviting* beings; most participants used terms like ‘benevolent’ (10) – sometimes associated with healing the participant (see *Helping*, **Role**) – almost equally conveying them as ‘benign’ (9), denoting their friendliness while not quite as profoundly loving. A diversity of 12 *Other dispositions* involved beings who were intensely ‘curious’ (4), itself notably always co-extant with the former two temperaments. For example, these participants describe entities with both such qualities:

“There was…a sense of being healed, a sense of [the observers] working on my physical and psychic body, eliminating toxins. Very benevolent, some curiosity from their perspective… They were curious to understand what I am, what I’m seeking… All very benevolent, looking out for me” (MP)

The next most commonly appraised demeanour, by five participants, was *Mischievous or Jestful*. However, as the inherent light-heartedness of the terms suggests, this does not preclude a warmer and playful character. This includes DD’s described above, and SH’s ‘fun and light’ was underscored by ‘an edge’ where there was ‘something about the presence that always feels slightly mischievous… Like it could take you somewhere really dark if it wanted to,’ and while JM describes that ‘there were hints that it could get dark’ he caveats this with that ‘everyone was in a good mood, for whatever reason! It was quite jovial.’

The only three encounters which had particularly challenging episodes (8% of total), labelled under *Fearsome or Menacing*, included two transient stages from RH. His third and last experience involved an emergence from ‘terrifying blanket’ patterns of hundreds of closing-in ‘hooded dark creatures, forbidding, quite forbidding, scaring me a bit’. Which was an apt judgement, given their eventually “saying something like, ‘Do not go there! Are you sure?’…like a test or something. ‘You sure, you sure? You can still get out of this!’”. It remains ambiguous as to whether they were displaying a more protective role to RH, or as part of some trickster-ish agenda (implied by the ‘test’ remark), akin to DD.

Often, as per the broader *Nature* of these entities, participants described themselves as ‘one with, or one of the beings’ (10) – one stating ‘the entities were my friends, I felt they were like my sisters, they were telling me that I’m like them’ (AF). RH’s third journey entailed an extensively tendrilled ever-evolving green-ish being of immense proportions (as did his second):

“Tough thing was that we knew each other so well, and I felt like I was one of them or something like that… I was much more connected to the body of it, the body and I were really close, like in an embrace of some sort, really close… I felt way more, much more like them than human.” (RH, Trip 3)

This refers to the same encounter in which he described such unity with the entities that they spoke and lived through him (see ‘possessing’, **Interaction**, Supplementary Material 1). Intimately dovetailed with this is that virtually all these reports of kinship also refer to a ‘familiarity’ (10) inherent in their relationship (that is, they are not familiar purely due to seeing them in prior DMT states), and two again describe the beings in a familial fashion as ‘seeming like a family [who] appeared around me for my lesson’ (ST):

“We were doing something that we have done before…We know each other. We are these things or something… They absolutely love us…Just wonderful, delighted, at home! It’s home” (RH, Trip 3)

‘Hyper-intelligence’ (7) was ascribed to the entities in several cases. RH (Trip 1) describes what he could only articulate as animal-like with circular pods on their heads:

“Yeah, hugely advanced…the difference in form, compared to this planet, that they were in just made me think this has to be a form of advanced [life]”

#### Communication and Messages: Entities as Instruments of Personal and Universal Insight

This super-ordinate theme encompasses explicit exchange of some form of understanding between the entities and the experient, evidenced by the majority of the beings. The *Mode of communication* was sometimes with the use of ‘dance or gesture’ (3), but for the most-part was purely ‘intuitive or telepathic’ (11), as RH (Trip 2) clearly conveys saying:

“I do not think it’s the ‘I’ that I think with that’s doing this at all, it’s something else. Because I often notice that I’m already communicating – I’m aware now, I’m effortlessly communicating with this entity… It’s not language, it’s just a knowing, just a complete knowing. I tried to tell them what I was doing at one point in English, but it’s just pointless!”

Listed in Supplementary Table 2, under *Messages received* are only the communications expressly verbalised by the participants and are by no means an exhaustive collection of all the subtle messages conveyed to each. These overt communications would be heavily associated with the aforementioned ‘teaching’ and ‘healing’ **Roles**; imparting to the subject of interpersonal guidance around ‘love for others and the self’ (5), entreaties for the person to ‘let go’ of suffering (3), ‘warnings’ (2) such as that ambivalently issued to RH, and another explaining ‘insights into the nature of the universe’ (1). The following, independent subtheme has been derived from this latter subtheme of universal insight:

The subtheme of ‘the Cosmic Game’ represents a constellation of profound communications expressed independently by five participants. Three of these had remarkably comparable messages; that the universe is a vast interconnected playground for beings to simply enjoy. AF is reproduced here, yet both her and SH were interfacing with a playful, flirtatious, feminine creature, functioning as both guide and teacher of such universal insight:

“Oh God, very very sensual. And this, like ‘it’s a game, its’ all a game’. They were playing with me, and they were like ‘come and play’. It was all a game, it was very very playful. Just enjoy… It’s like I’ve known them before, they were like ‘Yeah you know the game’, this is the relation. I know the game… They were showing me things. The main thing was this geometrical figure that kept changing shapes and colour. And they were…saying ‘Look what we can do, look what *you* can do!’. Very interacting, very interacting… These sensual females, they were like ‘Ok you wanna do this, Ok we are ready, whatever you want to do, we are ready’… They were telling me that I’m like them and I have to enjoy the pleasure of this existence, and life is fun, and it is all about playing and enjoy life and all that life has to offer… It was very liberating, and just like, let it out…just be… I had that sensation of just existing… I just opened myself…even the crying was like Yeahhh, crying, just Ahh, let it go!… It was showing me the things that exist. This exists and this exists, and they all had a pattern, they all looked like each other… They exist…in everything. We all exist in the same way. So there’s no separation of things… Everything already exists and is it out there”

A further super-ordinate theme, **Interaction and Behaviour**, encompassing its respective themes and descriptions, namely *Active Involvement*, 28%, and *Passive Activity*, 53%, has also been analysed (see Table 2; Supplementary Material 3). Owing to its close comparability to, and thus further refining of, the above theme covering the roles and functions of the entities; however, it has been inserted into the supplementary material (see Supplementary Material 1).

### Exploring Other Worlds

One hundred per cent did describe the concept of arriving at a qualitatively different space of consciousness or reality, characteristic of the so-called breakthrough DMT experience, and the major content of which is elucidated below. Smaller proportions variously reported more minor themes of *Breaking through the veil*, 22%, discovering their *Emergence into* a *novel reality*, 58%, and a *Navigation through* the *space* in which they now found themselves, 31% (see Table 2; Supplementary Material 3). For an elaboration of these, see the Supplementary Material (Supplementary Material 2).

#### The Scene: From Natural to Artificial Worlds

Entering into entirely different domains appears ubiquitous with these experiences, where the nature of this new world-environment took on myriad forms for participants: Weird worlds, yet betraying a familiarity, since within their transformed nature they may uncannily echo worldly experience (Shushan, 2018). *Natural worlds* recurred in 10 cases, where those pertaining to a ‘space-like environment’ (6) was most prominent. AZ saw himself ‘enveloped by a mystical black colour of the universe, inside of which there were an infinite number of geometric figures’, and SH, alongside the emergence of similar shapes, relates:

“I pulled back and it changed into a blackness, kind of like pyramids, quite angular shapes, but like black with almost like stars or something, there was some white or gold bits in there… these dots that could almost be stars, which felt tied up in the astrology in the Egyptian ancient wisdom”

Natural landscapes (4) which may be recognisably earthly, albeit a divinised mirror-image, included RH (Trip 1) and EM which shared an environ of living mercurial structures, and were again paralleled in their visions of gardens of biblical proportions:

“The beauty…is just so intense… I do not know what 5D looks like, but it was like that or more… A garden of extraordinary beauty, that [the animal-entities] were coming out of” (RH)

“Actually, it had gardens as well, because I was remembering there, like Oh you can definitely go to the garden and see some other stuff, it has rivers and everything. It was just like one of those palaces form ancient Babylonian gardens or something, it was beautiful, and every single bit of the wall – you know like mosques have all these patterns and all these beautiful glass like stuff inside, that everywhere, on every single little bit” (EM)

*Artificial worlds* comprised of six cases of vision-scapes possessing a particularly unnatural flavour, but most simultaneously expressed using natural imagery. This was observed before with FF’s icon-like creatures, which he again describes here as within a ‘mechanism’ of some description (2):

“Like conveyer belts, but within conveyer belts, they were everywhere… there was definitely a sort of sense of almost mechanical conveyor belts of psychedelic energy… Very sci-fi… how the space invaders moved across the screen, in very basic computer graphics…

But beautiful, it felt like the natural order of things… There was a purpose to it! Yeah, they were up to something… Like this whole thing was like the innards of a machine… but maybe your blood flowing through your system”

Visited by three participants were *Children’s worlds*. TM says of his second experience that he was clowning around in a ‘playroom’ (2), with ‘toys and entities and kids, someone just threw me there and said play with these entities’. The first words of GR’s interview, who was greeted by a sticker-like cut-out clown, were:

“It was literally like…everything was like completely shifted into another way of interpreting reality… into like, a circus sort of!… It was very ‘circusy’, because of the hilarity of it all!”

After asking what it was about the hilarious quality that transposed into a ‘circus’ (1), he replies, echoing his moving through the space *via* its own hysterically impossible ‘folding’:

“Because reality was just shifting – like you know those books, when you take one page and then they create like a thingy, a 3D object… Like that experience of a universe folding and unfolding, like that. It was just hilarious”

Interviewer: Like the hilarity of the cosmos being distilled into a children’s playful pop-up book that you were being shown through?

“Yeah, exactly! Exactly!”

Thirteen individuals expounded on, at least segments of their experience, being not so much well-defined worlds as much as a *Nebulous worlds*. One experient, OR (Trip 2), speculated he was witness to the etheric ‘fabric’ of the world around us:

“There was a time where I see like, atoms and scales and the matrix, like buzzing *Chh, chh, chh*… this was like you know, what’s the word, an ether…and it could just transpass [*sic*] me and everything… The fabric, like the fabric of what is this we see …this [gestures to room around him] is a manifestation, *not* an emanation… everything’s just wave or formless, and this [gestures again] is more dense you know, frozen part of all of this”

Various articulations of finding oneself amongst ‘gridwork’ (9) were most common here, alternately described as ‘not quite a honeycomb… lattice-work’ or ‘loads of intricate crisscrossing, like an amazing beautiful little spiderweb’. In both SH and GR’s very resonant experiences, there appeared a vast, devoid expanse, but one rich with potential in which objects were seen to materialise. GR’s lattice-like space represented a kind of cosmic template (2):

“I just did not exist anymore, it was just like raw data outside of me existing… In an infinite universe of raw information… [Like an] empty blueprint of the universe… Like this [points to mathematical paper] literally… it was a simple grid on horizontal, then a chalice there, but this was like one image out of a million [objects]… [It] was basically deeply knitted…like a fabric… So imagine this [sweater], but…with really big strings, but as if you’d make a huge cube out of it which was infinite”

#### The Contents: From Organic-Mechanic to Abstract Objects

As is the prolific nature of the content-full DMT journey, these other worlds are densely populated with a litany of articles and artefacts. *Organic objects* seemed to be the prevalent-most, aligned with the natural scenery (above) as being amongst the commonest, surveyed by 16 journeyers. For instance, ‘plants and flowers’ (10) stemmed from such simple images ‘of a tree, big kind of oak tree with a swing in it, and then loads of birds singing’ (JA), extending to more complex arrangements as AF’s floral, fractal encounter (much like BW):

“Some vase of flowers… And from inside there was this geometric shape, but they looked like flowers, like sacred geometry – and what it was really, was…a male entity in front of me… showing me a flow of energy, shapes, colours – flowers coming out his solar plexus… Everything he was throwing out of his body was related to one another and linked by shape and meaning”

Imagery of a ‘cellular or sub-cellular’ nature was also recounted (4), where two such experients expressly noted DNA. EM upon entering her ‘impossible… cube but hexagonal’ (‘hyperdimensional structures’, below) was privy to ‘a cell or something…and loads of organelles… So it’s like a 3D hexagon, with an interior made out of cell-like structures, like DNA strands’. TM, during his third time, upon being swallowed by a child, couched his double helix in the following terms, very analogous to the ‘organic-mechanic’ aesthetic reported by FF which he posited as a view inside biological machinery:

“I saw very strange DNA, but wasn’t DNA that I know, it was made by… It was like a chain, and in the middle they had…definitely a sphere with, they were not symbols, it was made of something, seems mechanical… I remember very well a sign… It was intense red these things, and all of these were functioning somehow, like a mechanism, not a biological one, more mechanic… And everything with a note of cartoony. I wasn’t sure if I was seeing inside a biological being of someone”

Descending ever further in scale, the ‘molecular or subatomic’ (3) revolves around ‘seeing all these different molecules… just everywhere were just collections of molecules and geometry’ (JA), or in RH’s case (Trip 3), initially speaking of a building, ‘like the girders inside, the structure inside something’, yet qualifying that it ‘looks like in chemistry, the shapes of atoms’. The elaborate-most exposition of this idea comes from part of ST’s depiction of his hyperactive hypercube (see *Geometric objects*) – also verging on interpretations of a metaphysical ilk (*Meta-narratives*, as discussed in the subsequent paper of DMT’s effect on the self):

“When I stepped into it, it seemed like [the hypercube] was a fundamental thing in reality, almost like [the entities] took the smallest thing on the Planck scale for example…to maybe represent the scale, then you blew it up to show you that this *is* the way things are on the tiniest layer… This is what builds up everything!… It’s like a computer screen or a newspaper, where they have a big image and then they have a close-up. Like a microscopic image of this whole thing and here’s what’s going on there…

It seemed the Borg thing [i.e., the hypercube] came in 3-dimensional space…everything else was shimmering…because it was in more high detail… I can actually see the finest definitions of this… It seems like I’m looking at this and I can actually see it inside-out almost, I can see the whole thing, like on a molecular level”

ST had also referred to his tessellating tesseract, in its rapidly rotating configurations, as like the ‘Autobots’ (of the *Transformers* franchise), thus representing one of eight instances of *Technological objects* appearing. A sense of ‘mechanics’ and curious ‘devices’ were sometimes evident (4). Both AN and JM turn to cog-like imagery, the former describing her pressing up to her DMT partner’s back as ‘a fusion of the spines and all the energy around them, like turning into one another, like cogs, like kind of twisting in… that central column entwining’

Lastly, ‘satellites and spaceships’ (2) were volunteered, where comparably to ST’s hypercube framed as that from ‘The Borg’ (of *Star Trek*) – so in precisely the same way does BB paint his hypercubic craft as:

“you know, the Borg, and their cubes…it’s a big spaceship and it’s a cube, obviously not grim and grizzly like that. But a big cube that has a lot of detail within it, almost with little cutaways, and each cutaway is its own 3D space – and the whole thing, this 3D shape rotates, sort of spins, not slowly, almost as if some really cheesy American documentary is trying to make everything look razzmatazz and sort of sex it up, like *dumdahdahdumm*! Everything’s moving, almost as if it’s trying to catch your attention”

Such ‘hyperdimensional structures’ (6) recurred with noteworthy frequency, subsumed under *Geometric objects* presented to 16 experiencers, where five of the six such hyper-structures being cubes of multidimensional proportions. Here, DD fleshes out his feminine-faerie as puppeteering him through:

“a cube…the multiple dimension aspect of it was that she- it started off like 2D then it sort of slipped into this cuboid which then fractalated…

Interviewer: …a cuboid, but more than 3D?

Oh mate, you fucking- absolutely!… as this cube sort of splintered off, and I was able to sort of look around the other facets of this cube if that makes sense… it was weird- it’s such an understatement innit [*sic*]. So as this cube- she’s lured me, and the cube is fucking sort of disintegrating in multiple dimensions at the same time… but that scene [of the woman and child] was the mirror in one of these shards of this cube that had just splintered…

The female entity that I’m quite familiar with, she then came back in this sort of multi-fucking-splintered multi-dimensional thing, and was giggling… the 2D square then broke into this sort of hyperdimensional cuboid fucking thing that just then exploded, and I sort of kind of went into it… then it just went fucking- accelerated into super crystalline- I remember looking at it and appreciating how unbelievably fucking impossible those lines are and that situation”

Virtually the only other geometric object, dissociable from the perhaps pervasive geometric patterns in DMT, appeared to be ‘spherical shapes’ (7). For example, TM’s (Trip 3) earlier organic-mechanic DNA mingled with a sphere, and others express similar impressions that ‘there were definitely balls there as well…like spinning balls amongst it’ (DD).

*Symbolic objects* occurring in six total cases were signalled perhaps most vividly by TM’s experiences, especially his second immersion. For instance, in terms of ‘symbols or signs’ (5), many such signs to him represented medicine, alongside a ‘kind of cross or something in the middle, then borders like an arms crest’ (TM 2). This echoes his first trip where ‘around that pill were floating four or five symbols’ (a shuriken-like cross, large tree and swirls).

Similarly, ‘ancient language or hieroglyphics’ (3) were repeatedly observed, from ‘very, very Aztec…patterns’ (RS), to TM’s (Trip 2) toys endowed by his clowns which:

“appeared like, Egyptian, maybe not, but hieroglyphical writings, that was a kind of paper, but it was floating like a papyrus but… Some of them were pictographic, some of them were hieroglyphical writings near [the toys]… Some of the toys looks like a Greek vase…I saw Greek motifs on some”

Again, TM’s (Trip 2) experiences continued in highly symbolic form, reporting the classic digital ‘raining code’ from *The Matrix* trilogy, as had one other journeyer – which was, for TM, manifested alongside a trinity of black holes (‘space’, *Natural worlds*):

“I saw codes, a lot of codes, walls infinite with codes. At one point, very fast, to form three black holes in those codes that were flowing all over. It was like The Matrix code, it was even green. Very, ‘MS DOS’ code… alien language, I could not recognise those… crawling down and up, and some of them were attracted toward those black holes

Interviewer: So they were bending in space toward the black holes?

Yeah, yeah, yeah! And after that I could see…other kinds of codes, different language…and this one was somehow more complex, and it came like a typhoon over me… So all my visual spectrum was codes, and they were green, and it was a black space. Bright green, neon green”

Apart from the aforementioned nursery and children’s book scenery, TM (Trip 2) is most representative of the three case’s *Infantile objects*. He here tries to comprehend his gifted toys, the gyrating gadgets which:

“were somehow alive, continuously moving and changing shapes, and colours… Some of them had fluffy texture, some of them were mechanic. Some of them some strange, very strange material I cannot define… at the second look, it changed very radically the form, it was something undefined, because morphing continuously…

these bubbles transformed into the toys – they were very coloured, the colour became cartoonish somehow. They were starting- it was a screen-saver of Windows 98 with these kind of orbs changing shapes…from bubbles into the toys”

At least 14 experients offered up myriad *Miscellaneous objects*, perhaps more arbitrarily positioned amongst the vision. However, additionally to the snake-haired experimenters of TM (Trip 1; and several other snakey entities) – who also added that ‘snakes I think they were one…or two of the symbols’ – many other kernels of imagery had decidedly ‘serpentine or cyclical’ spins to them (7). Again, in his second session, TM rounds off his description of his intricate symbol-adorned toys, redolent of the medical insignia of the serpent-entwined staff, by saying:

“you know, the Chinese carnival, those kind of motifs, they were very similar… Some of them were boxes made by kind of dragons that were moving in that form…for example, the dragons had the crest and everything, red, blue, green, very colourful… This sign morphed from that dragon, Chinese dragon…because at first that dragon was moving on that toy like a snake, and protecting that toy, and the end of the tail it had a sign… for me it looked like a medical thing”

#### The Quality: A Mixture of Textures and Transformations

Pertaining to the kind of visual texture to the general world-space and its constituents – at least three of the five tesseract-structures were explicitly qualified as *Transforming or exploding*, including BB’s ‘rotating’ or ‘spinning’, and DD’s impossibly ‘fractallating [*sic*]’ or ‘splitting’ cubes. These transforming configurations have also been visited in four other instances, like during TM’s (Trip 2) clockwork-clowns’ shifting gifts.

Fifteen participants noted aspects aligning more with the environment’s *Synthetic textures*. Perhaps more *semi*-synthetic, most evoked was the (by now familiar) peculiar hybrid-aesthetic of ‘organic-mechanic’ (5), such as with TM’s (Trip 3) DNA-complex he ‘was looking to see if it was molecular or mechanical – and it was a mix, definitely!.’ Again though, much of this *synthetic* quality in general pivots around the hypercube phenomenon – where both BB and ST analogised theirs as like that of ‘the Borg’, which is a contraction of ‘Cyborg’ (and in turn, of cybernetic organism). BB elaborates:

“There was a woody quality to it. Bark-like. It’s never pure geometry. If you have got [at] one end of the spectrum, artificially, a 1990s fractal poster, to a tree and its fractal patterns – it’s sort of somewhere in the middle”

Equally as many individuals reported a ‘cartoony’ or sometimes ‘animated’ quality to things (5). Resonating again with BB’s high-dimensional cube – also depicted as ‘razzamatazz’ (1) – ST relates that ‘it’s the Borg in cartoon… Imagine a *Rick and Morty* version of it. With every single component of it moving about and in different colours’, thus evoking another animated, space- and interdimensional-themed TV show. Finally, scenic finishes such as MP’s ‘super holographic, very digital looking’ (3) world occasionally arose, which was also inhabited by a hypercube. Although this evinces artificial feelings much akin to BB’s or ST’s CGI-like, technological cubes – ST considered his as part of the substructure of reality, and MP in fact also qualifies his as ‘hyper-real’.

## Discussion

The present analysis from high-dose (>40 mg) smoked DMT in naturalistic settings yielded all 100% of the 36 experiences to include either contacting representations of another being, or emerging in a different environment (virtually always co-occurring), of some nature. The experiences of ‘breaking through’ to other immersive worlds, that are hyper-vivid, intricate and impossible, as well as interacting with other beings, apparently sentient, independent and familiar, have been retold from the original and revitalising human experiments with DMT, to the only other modest thematic analysis and very recent online studies of the encounter discussed below. Such experiences have been thoroughly reconfirmed by the present analysis. Indeed, the frequent overlap of, often specific and nuanced, content between participants in the present study is testament also to the interesting internal consistency of the DMT experience.

### Parallels With Major Studies of the DMT Experience

Shanon (2002) categorisations, though pertaining to ayahuasca, also very closely reappeared in the present analysis, such as ‘soul-flight’ through the other realms, which may similarly be of natural or heavenly landscapes; ancient or magical cities; seeing Earth, outer-space voyages; amusement parks and circuses; mythological imagery, scripts and symbols, mathematical formulae, and biological evolution. As per the native populace of these realities, while animals were comparatively uncommon in this analysis, snakes, and serpentine beings and imagery, were also the commonest animal-motifs met. Saliently too, all the (albeit few) known human beings met were deceased – DMT echoing ayahuasca as ‘the spirit molecule’. While shamanic-like spirit transformations were not directly reported, several participants did describe feeling ‘one’ with the beings encountered, and one transformed into plant-life (where two participants reported human-animal hybrids). Other strata of Shannon’s phylogeny were again mirrored, including mythological-types such as other animal-hybrid beings or faerie-natured entities, ET or alien-types (like serpents, octopoids or insectoids, grey-like beings or Navis); beings divinely angelic in character and function; though entities of ‘demonic’ dispositions were barely encountered, where one subject reported a succubus-like figure and another a multi-eyed entity congruent with many religious depictions of beings of death, also elsewhere reported with DMT (Luke, 2008). Specific deities of particular religions or mythologies were *never* explicitly identified in this analysis – though overlapping qualities were certainly discussed (such as *Shakti*) – perhaps related to the bracketing (inherent to micro-phenomenology; Petitmengin, 2006, 2017), similarly encouraged in the semi-structured interview (see Materials and Methods).

The content analysis of discarnate DMT beings by Lyke (2019) – subtitled ‘Not everyone gets machine elves’ – elicited highly similar findings to the present paper’s results. She identified that 75% of reports included at least one form of entity where 37% described more than one, though the present study in fact found 19% to have at least one and 75% with more than one. When using only her specified lists for comparison, comparing Lyke first and the present analysis following, entities were highly significantly more likely to be female in Lyke versus twice as common in the present sample, and poorly defined/featureless (29 vs. 22%), as well as humanoid beings (22 vs. 25%) the commonest in both. Though more difficult to make direct correspondences, due to the splitting into nuanced and various subthemes in the present thematic scheme, Lyke listed divine (10%) followed by alien beings (8%) as the next most common, where those entities with obvious physical or behavioural attributes of an angelic or alien nature would also approximately follow next here (however the latter, e.g., Octopoid, Grey/Mantis etc., more prominent than the former, e.g., Benevolent, Beautiful – though, the categorical alien-angel distinction likely lies only in *appearance*). Lyke’s categories then follow with elves/faeries (7%), animals (6%), then mechanical and geometric objects (6%), where similarly, all such entity types were found in this analysis – albeit reversed in frequency in the present (i.e., 3, 11, 8–22%), though here with elfish creatures defined only by appearance versus nature (e.g., Mischievous, Childish). Identically, voices (4 vs. 3%) and faces (4 vs. 6%) alone were the least most likely type.

Lyke and the present analysis again closely converge in relation to interactions with the encountered beings. All of her thematic labels were evident in the present framework (except for ‘questioning’, 3%) and the prevalent-most theme being ‘showing, teaching or guiding’ (25%) – equivalent to the roles of ‘presenter’ (28%), ‘teacher’ (25%), and ‘guide’ (14%) in this analysis. Similar order of prevalences included Lyke’s ‘love’ as the 3rd most common (9%), with ‘benevolence’ being the commonest demeanour in this analysis (28%); ‘observation’ was the 5th most common (7%), with ‘watching’ being the commonest interaction here (25%); middling themes included ‘excitement’ (9%), ‘reassurance’ (8%), or ‘play’ (4%), which may correspond to similarly middling ‘jovial’ nature (11%), and ‘soother’ (14%) and ‘playmate’ (14%) roles here. ‘Power/control’ (3%) and ‘sexuality’ (3%) may correspond to the comparably minor ‘orchestrator’ role (6%) and ‘sensual’ demeanour (3%) here. Particular discrepancies however include Lyke’s ‘hostility’ as being the 2nd commonest (10%), while the equivalent ‘menacing’ beings (8%) in the present study were certainly the *least* frequent – most likely a function of our participants’ being accustomed to DMT and in a quasi-controlled space amongst quiet researchers (suggesting, even at breakthrough levels, DMT’s sensitivity to ‘set and setting). ‘Reminding’ was Lyke’s least common (2%), whereas, though nebulous, virtually *all* themes within ‘communication’ herein, of insights into relationships and the universe (3–14%), often had an essence of reminding inherent, and indeed familiarity with the entities was the commonest ‘nature’ herein also (28%). While Lyke reports no interaction (10%) as joint-second commonest, the vast majority of encounters here exhibited some role or interaction (14% minor/no engagement).

Parallels between this study’s results and those of Davis et al., 2020b DMT entity survey include many of the same parallels with Lyke (2019). Though, one important difference between the present analysis and Davis et al. is that the latter employed mostly fixed, pre-categorised responses, meaning fewer and pre-conceived options versus the free vocabulary of this study’s participants. Davis et al. most frequent selections included ‘guide’ (43%), ‘alien’ (39%), spirit (39%) or ‘helper’ (34%), where in the current analysis the Helping roles (53%) incorporating ‘guide’ (14%), as well as alien appearances (e.g., Octopoid to Grey-like, 6–11%) were certainly amongst commonest – though ‘indigenous’ or ‘succubus’-like spirits were among the least reported (3–6%). Davis et al. next most frequent included ‘angel’ (16%), ‘elf’ or ‘faerie’ (14, 8%), or ‘religious personage’ (11%), where again, angelic-resembling beings would also approximately be some of the next commonest types here (e.g., Benevolence, 28%) – though elf- or faerie-looking beings were of the *least* common (3%), and specific deities were not explicitly identified. Then decreasingly prevalent were ‘clown’ (6%) or ‘demon’ (6%), where ‘menacing’ beings were certainly the utmost least major demeanours herein (8%) – though, clown-like characters were actually one of the dominant (11%). ‘Plant-’ (10%) and ‘chemical-spirits’ (8%) were reported in Davis et al., where though highlighting shamanic-type motifs as found in our study (which Lyke had not), these particular descriptors were lacking here, as were ‘animal-spirits’ (7%; except animals, 11%, and therianthrope-types, 6% here). To be discussed further in a subsequent paper relating the present analysis to near-death experiences – pertinently, both Davis et al. (1–2%) and this study found the deceased to be amongst the very least reported.

With regards to participants’ interpretations of entities’ natures, the majority of Davis et al., 2020b respondents endorsed ‘benevolent’ (78%) and ‘sacred’ (70%), where again ‘benevolent’ (28%) was the commonest nature in this study; ‘all-knowing’ (38%) was one of the next most selected, and ‘hyper-intelligent’ (19%) also being amongst the commonest natures here; and similarly ‘malicious’ was the least common (11%; contrary to Lyke), as it was here (8%). Familiarity was ‘slight’ for Davis et al., whereas over a *quarter* described the entities’ familiarity in this study – though notably about two thirds of the Davis et al. sample referred to their first encounter, versus the usually multiple past encounters in the present study (though again, almost all entities herein were novel, thus familiar for other reasons). Davis et al. also included having agency (54%), positively judgmental (52%), eternal (42%), and petitionable (23%), not directly coded here. ‘Distrust’ of the entity was reported by Davis et al. (10%), which may well align with the trickster-like beings of the present study (11%), those mischievous characters evoking ambivalence.

Davis et al., 2020b communications (‘Message, task, purpose, insight’ and ‘predictions’) transmitted to 69% of respondents were also similar to the present analysis’s ‘communications’ theme; 39% of participants articulated messages though many more qualifying for communication. Namely, Davis et al. ‘personal insight’ (22%), ‘love’ (16%), ‘reassurance’ (10%), ‘interconnectedness’ (7%), ‘death’ (7%), ‘knowledge’ (6%), ‘afterlife’ (2%) – also, crudely, matching in terms of decreasing prevalence – correspond to this study’s messages of ‘insight into world’ incorporating ‘cosmic game’ (14%), ‘love for self/others’ (14%), and ‘letting go’ (8%), as well as the roles of teaching (25%; and content of said lessons) and soothing (14%), and benevolent demeanours (28%). Specific ‘tasks or purposes’, as well as almost all ‘predictions’ about the personal or global future however, were decidedly *absent* herein. The vastly telepathic, then visual mode of communication, was also shared in Davis et al. (74, 40%) and here (36, 6–8%) – though verbal (26%) and tactile (17%) were not present here. The encounter is finally reported as ‘hyperreal’ by the majority (81%), including causing significant ontological reorientations (80%) – as also identified in this study (mainly former; though reported in the subsequent paper on effects on the self).

### Echoes With Other Extraordinary Human Experiences

While *all* participants volunteered many basic features considered endemic to the near-death experience syndrome, as similarly found with a recent psychometric study comparing items of the NDE scale (Greyson, 1983) between near-death and DMT experiences (Timmermann et al., 2018), the similarity remained superficial in the present qualitative analysis of DMT content. To briefly short-list the generic near-death themes coded in the remainder of the wider DMT analysis (not expressly reported here) which may correspond to the most frequent structural features across NDEs (Charland-Verville et al., 2014), these included – deep positive mood, bodily dissociation, bright light(s), deceased loved ones (albeit comparatively low), explicitly god-like beings or light-beings, and time transcendence. Also coded and aligning to traditional NDE-themes were tunnel-like structures, translocation elsewhere and a sense of dying.

Possibly the most comparable experience to the present DMT report in content and not only structure is that of alien abduction. Many such as Mack (1994, 2000) have thoroughly documented this phenomenon, where Hancock (2005) has already found considerable overlap between them and ayahuasca and other shamanic experiences (and faerie folklore), and Strassman (2001) emphasises the alien abduction-DMT resonance. Here, very many themes are evocative of abduction, such as the entity roles of ‘presenter’, ‘orchestrator’, and especially ‘experimenter’, and the appearances of ‘insectoid’ and ‘serpentine’ beings, and particularly the ‘grey- or mantis’-like beings who although not reported in Davis et al., 2020b seemed present in two participants’ experiences here (admittedly, without the short stature or grey colour as per the former). An ‘intelligent’, ‘powerful’ and often ‘familiar’ nature; mostly ‘telepathic’ and ‘visual’ mode of communication, propensity of simple observation, and notably often ‘curious’, sometimes ‘urging’ demeanour were also highly reminiscent of abduction lore. The two experients in this study, explored variously above but revealed further in a subsequent paper, incorporating such themes and most resembling the phenomenon, would be TM (Trip 1) and MP. Despite suggestions of the laboratory environs of Strassman (2001) producing the abduction-esque scenarios (Luke, 2017; at least regarding medical-type procedures), this naturalistic study still yielded resonant themes in at least these two (albeit expectation may be a factor).

In a similar vein, parallels between faerie folklore (of the British Isles) and DMT experiences have been explored (Rushton, 2016, 2020), and compellingly demonstrated with ayahuasca (and other shamanic) experiences (Hancock, 2005). Those themes in the present study which are redolent of the ‘little folk’ have already been touched on above (in the reports of TM, DD and JM), also encompassing such appearances as ‘faerie’- or indeed, more commonly, ‘clown’-like; demeanours of ‘playful’, ‘childish’, ‘laughing’ or ‘bounding’ around, and not least ‘dancing’ entities (akin to the quintessential ‘fairy dance’ in the lore). The faerie phenomenon itself, however, has been cogently argued to be a progenitor of the contemporarily stylised alien abduction experience (Vallee, 1969; c.f. Hancock, 2005), where both converge, for example, on a spiriting away by little humanoids (often with large black eyes) leading to experiences of subjugation, learning, or (not evident here) breeding. More precisely, for the purposes of the present DMT comparison, faeries/aliens have also been depicted as being ‘trickster’-like, with ‘mischievous’ or deceptive inclinations – and as ‘hyperdimensional’, with such origins in hyperspace indeed speculated by others (Evans-Wentz, 1911; Jacques Vallee, 1993) – as have the DMT entities herein. Perhaps an exemplar of this elf-alien-DMT entity triad would be DD, with his childish (but powerful), faerie-faced, hyperdimensional trickster luring him through a tunnel and focusing his attention on screen-like shards. Importantly, a further prong to this trinity may be the spirits of the dead, taking us closer to the near-death experience. While the ‘*spirit* molecule’s evocation of the dead was minorly revealed in the current report, faerie folk were considered entangled with spirits of the dead (Evans-Wentz, 1911) and the materialisation of the deceased may be more common than appreciated around alien abduction (Streiber, 2022).

Shamanic flights to, and animistic encounters within, other worlds has also been shown to have substantial consistency, not only with ayahuasca, but with DMT experiences (Winkelman, 2018). Briefly, some themes elicited in the current study and such shamanic experiences include entities enacting ‘healing’, ‘teaching’ and ‘possessing’ functions and actions, who may be in the form of spirits; the observing of realms composed of ‘lattice’-patterns or other geometry, viewing inside the body, and molecular structures like ‘DNA’ or ‘serpentine’ imagery (e.g., Narby, 1998). Deep qualitative resonances between shamans’ journeys and both faerie and modern abduction lore have also been evidenced (Ring, 1992; Hancock, 2005), in turn resonating with DMT reports herein. These entail the ‘soul flight’, communications intended for ‘insight’ into the nature of the world or relationships in it, such as the spirit realm or interdependence; as well as ‘therianthropic’ beings, or even the few cases of sweets in cellophane (RH; as offered by those in the ‘spirit world’), a ladder toward light (AN), and a bible-like book (HV). The participants RV, JA and HV are mostly comprised of such shamanic elements – the latter two being the only participants who actively engaged with DMT in a ritualistic context.

### Potential Neural Mechanisms

Of course, such echoing between DMT, near-death, abduction and shamanic phenomena could indicate a role for the endogenous psychedelic in each (qualitative evaluation of this regarding NDEs will come from a subsequent analysis). Alternatively, similarities may be owing to converging downstream mechanisms, such as default-mode network (DMN) disintegration (e.g., Winkelman, 2017), considered the gross neural correlate of psychedelic action by 5HT-2A agonism. For example, *salvia divinorum*, a Mexican shamanic herb and atypical psychedelic generative of disconnection of consciousness to immersive spaces, though stimulating the kappa-opioid receptor it similarly reduces static functional connectivity in the DMN (Doss et al., 2020). Neurophysiologically, such radical perceptual restructuring and ‘world building’ may be owing to collapse of alpha-beta wave, and dominance of delta-theta wave (Timmermann et al., 2019; Pallavicini et al., 2020) and forward travelling wave activity across the cortex (Alamia et al., 2020; Gallimore, 2020), evidenced at least by DMT.

In terms of the entity encounters analysed herein, and characteristic of many of these other altered states, Winkelman (2018) elaborates that the relinquishing of cortical control by structures like the DMN over hierarchically lower brain systems may release innate neural modules, key of which may be a ‘hyperactive agency detection device’ (HADD; linked to the faculty of mentalising), evolutionarily conserved to identify probable social agencies, and especially human-like agents in humans. This is also congruent with the anthropomorphic natures and behaviours to the present studies’ entities, including the most-endorsed ‘humanoid’ appearance. Timmermann (2019) has presented preliminary results from functional magnetic resonance imaging (fMRI) of the DMT state showing decreased connectivity in several intrinsic networks, including the DMN (consistent with previous psychedelic imaging data), as well as electroencephalographic (EEG) results suggesting inhibited alpha and elevated theta power, the latter across the temporal lobe (also exhibited by many dream-like states, Harris, 2007). Such data also appear consistent with recent findings of baseline temporal (and frontal) theta oscillations inversely correlating with mystical experiences of unity and transcendence (Tagliazucchi et al., 2021), where presumably such a non-dual subjective state may preclude the dualistic encounter with a non-self ‘other’.

As per Timmerman et al.’s fMRI findings, similar DMN disorganisation is concurrent with alpha power attenuation over the posterior cingulate cortex, a key node of the network, under psilocybin (also correlated to ‘ego disintegration’), where higher cortical alpha is otherwise thought to carry forward predictions (Muthukumaraswamy et al., 2013; Carhart-Harris et al., 2014). There is also evidence pointing to such predictive processing operating when exposed to biological motion and traits, thus applying to theory of mind (ToM; inferring about the internal state of another; Koster-Hale and Saxe, 2013). In this way, theory of mind may be disrupted by DMT with the brain struggling to make the best prediction resulting in inappropriate interpretations of social agency. Regarding the above temporal EEG findings under DMT, it is likely that the release of the top-down constraining by the PCC (and DMN generally) of the medial temporal lobe (mTL) disinhibits the inherent driving activity of the latter over the former, with such freed intrinsic activity giving way to the psychedelic ‘primary state’ (Carhart-Harris et al., 2014) which may include as part of it ToM processes. For instance, mTL epilepsy patients show significant impairments in ToM (versus other epilepsy and schizophrenia subgroups; Broicher et al., 2012; Okruszek et al., 2017). Additionally, both the superior temporal sulcus and temporo-parietal junction are considered part of the ‘mentalising network’ (Aichhorn et al., 2009), where the latter as well as the medial prefrontal cortex (possibly unique to the human brain) are activated during social inference (Frith and Frith, 2000) – which, as such, may be similarly implicated under DMT.

Despite the above putative mechanisms, it still presents a challenge to account for the entity experience, especially given their manifestly baroque and highly profound (and reportedly ‘hyper-real’) nature evident by the present study. Generally, the novel statistical associations of the disparate, normally non-correlated, neural networks afforded by the intrinsic network disintegration and concurrent global network desegregation under DMT (and psychedelics generally, e.g., Carhart-Harris et al., 2016), may integrate into it the activated substrates of theory of mind in a broader synthesis, where such ToM activity may now ‘drive’ other neural systems such as the occipital and association areas, and the resulting subjective visual and social content is simultaneously available to conscious experience. This is also echoed by the ‘phantasy’ mode of consciousness, serving to construct a storyline-like narrative embellished from the data presented to awareness (Horváth et al., 2018; Winkelman, 2018). That entities may be an end-stage result of the integration of the brain’s disturbed capacity to accurately predict patterns in ambiguous stimuli and an aberrant agency detection, alongside the complex visual, often kinetic, fractal-geometric, imagery presented under DMT, may be consistent with the 8 participants herein reporting the beings to have a geometric flare to them. This said, this appears to be more the exception than the rule out of the 36 interviews analysed.

Consistent with Laughlin (2000) model of the Jungian archetypes as being encoded into evolutionarily conserved ‘neurognostic structures’ in the brain, Stephan Szara, the discoverer of DMT as a hallucinogen, himself recommends looking for the origins of these entities ‘right in the brain’, yet ‘deeper than conscious memories’, further elaborating that such archetypes may be:*

“…stored in neuronal connectivity patterns early in development. What DMT might do… [is] let the Default Mode Network release the stored images and symbols into the perceptual system” (Gallimore and Luke, 2015 p. 11; Szára, 2014).

### Therapeutic Potential

The fact that the DMT experience is clearly evocative of all the above classifications of experiences also has central implications for its therapeutic potential. For instance, the near-death and abduction (or UFO) experiences are shown to increase ecological connection, spiritual worldviews (Ring, 1992; Groth-Marnat and Summers, 1998; Mack, 2000) and decrease death anxiety (Greyson, 1992; Mack, 2000; Bianco, 2017), while NDEs also foster connection to oneself and others (Groth-Marnat and Summers, 1998; Van Lommel, 2002) – all of which have been shown or proposed to mediate enhanced wellbeing in psychedelic settings (Watts et al., 2017; Kettner et al., 2019; Davis et al., 2020a; Moreton et al., 2020). Additionally, as supported by Davis et al., 2020b, the interpersonal and transpersonal insights received during DMT entity communication in this study suggests therapeutic capacity, in light of various studies indicating psychotherapeutic effectivity being attributable to psychedelically-inspired psychological insight (e.g., Gasser et al., 2015; Belser et al., 2017; Watts et al., 2017). Similarly, ontological implications of encounters with seemingly other-worldly beings who are hyper-intelligent, familiar and sometimes omnipresent, may be associated with elevations in psychological flexibility, which have in turn been evidenced or suggested as a therapeutic mechanism of the psychedelic experience (Davis et al., 2020a; Watts and Luoma, 2020).

All this said, although the vast majority of the encounters amongst seasoned DMT users herein were extremely positive, for less prepared and more vulnerable naïve and/or patient populations these same psychological ramifications related to ontology and the sheer intensity could well be challenging and yield adverse effects. Similarly, the precise relevance of the present study’s resultant experiences, using such experienced users in their habitual settings, thus with likely differing subjective content, for any therapeutic application of DMT with naïve populations may be undermined. Even so, there is extensive anecdotal evidence of the same specific themes recurring even amongst first-time DMT users (Luke, 2017), the unique richness of the content elaborated in the present study should still be of significant guidance to any party involved in DMT’s therapeutic research or use – and while experiences herein may be less challenging due to home environments, the presence of guides or therapists in therapeutic contexts should also be beneficial.

### Distinguishing Features of DMT

It seems that there is virtually no single experiential feature of the above thematised DMT experience which cannot be found in either the alien abduction, faerie lore or shamanic, especially ayahuasca, experience. However, there may be a select few which may be more unique to the DMT trip itself (possibly with ayahuasca, given DMT as, arguably, the major constituent). For instance, beings here described as clowns or clown-like (though perhaps finding their analogue in the faerie folk), or otherworldly environs as playpens or circuses – for instance, both articulated by GR; as well as entities with a decidedly mechanical or indeed an organic-mechanic quality to them (though UFOs also, for instance, have actually been described as having biological (Kripal, 2022) or animal-spirit dimensions (Luna, 2022)), or realms expressed as the inside of a ‘mechanism’ – again both expressed by FF. The repeated phenomenon of the hyperactive hyperdimensional cube (DD, ST, AF, BB, MP) may also be considered a preferred trope of DMT, as well as the raining code iconic of The Matrix (TM Trip 2, HV); as might the ‘razzamataz’/garish, retro game or even holographic-digital style. In short, prototypically infantile, synthetic or, unsurprisingly, psychedelic imagery. The current report’s cellular-molecular or skull-skeletal imagery are not typical of faerie or abduction literature, though may well be found in certain shamanic contexts (including ayahuasca).

### Limitations

While there have been a number of improvements by the current study on previous studies in their analysis of the DMT experience, not least its focus on content, prospective nature and in-depth semi-structured interviews, various limitations are still present. For instance, similarly to, though improving on Lyke (2019) and Cott and Rock (2008), over three quarters of participants were male, and roughly equalling Davis et al., 2020b slightly over 80% were Caucasian, thus decreasing generalisability to the general population. However, Caucasian males are the main DMT using demographic (Palamar and Le, 2018). The present report also involves an improved age-range (23–58 years). Additionally, the present sample were affected by a degree of self-selection, imposing certain selection bias, given participants’ likely volunteering due to typically experiencing more elaborate, meaningful, and even positive DMT trips. A stringent screening procedure was also employed, involving the inclusion criterium of at least one breakthrough DMT experience as well as other DMT or analogue experiences, meaning that participants were not naïve, usually having vary many past trips (see Table 1). This is a salient point, given the present study’s focus on experiential *content*, and content’s sensitivity to psychological history (Hartogsohn, 2016). More novice users may well have reported qualitatively different experiences, for example as suggested by Davis et al., 2020b finding that most respondents reported their *first* entity encounter as their most memorable. Such a narrowed sample is tantamount to the recruitment of a specific ‘subculture’, characterised by exposure to and interest in certain media, both fictitious and that regarding DMT research and ‘lore’ – which may well prime participants’ ‘sets’ and expectations. At least one way this may have contributed to the resultant phenomenology is the comparatively high rate of entity encounters in the present study (94%), contrasted to Strassman (2001) finding approximately half of the *high* dose participants reporting this main feature (Luke, 2013), only very few of whom had previously taken DMT. How this may have affected the content itself is very difficult to untangle, even if comparing against a hypothetical (and improbable) study amongst entirely psychedelic-naïve individuals. This said, a key boon of using such ‘psychonauts’ is their deeper acquaintance with the DMT-space and lack of ontological shock expected of first-time users. This was not only a matter of ethics in avoiding unpredictable and possibly alarming reactions, but would have allowed a calmer, attentive and exploratory stance during the experience, thus returning having extracted, and able to articulate, more of its phenomenological essence. In a similar vein, the majority of the sample’s nationalities being British (with some different ethnic descent), and most of these White British (see Table 1), may also have shaped the content of the experiences presented here. While a comparative analysis of the content differences between nationalities (or ethnicities) is beyond the scope of this study, future research would be of interest to illuminate this contextual sensitivity of DMT experience.

Finally, as to the relative control of the present study – the substance used was not tested for its genuinely constituting *LG-DMT*, nor for purity, and was always of plant-extract as opposed to laboratory-grade synthesis as used in the only two laboratory studies to date (Strassman, 2001; Timmermann et al., 2018). In spite of this, doubt can be minimised given that participants were seasoned DMT users intending breakthrough experiences attesting to the quality of the sample, as well as the researchers’ understanding of the appearance of the substance, and not least the resultant phenomenology of participant experiences being highly concordant with other lab. Studies’ reported DMT experiences – as emphasised in the above discussion. Additionally, the setting for each participant was not a closely controlled laboratory environment, and as such the different physical contexts between participants may well have influenced the resultant experience (Hartogsohn, 2016). This said, the naturalistic nature of the study meant participants undergoing the DMT experiences in their regular environment (own homes or gardens) with only researchers present, thus introducing a level of standardisation. Also, the uniquely stimulating setting of lab. Environments, though more homogenised, equally present a host of experiential primes which may have limited such studies (Luke, 2017). In this way, this degree of regulation of substance and setting, made possible by the present field study, was markedly improved compared to other major DMT studies using only online surveys or forums (Cott and Rock, 2008; Lyke, 2019; Davis et al., 2020b).

## Data Availability Statement

The raw data supporting the conclusions of this article will be made available by the authors, without undue reservation.

## Ethics Statement

The studies involving human participants were reviewed and approved by University of Greenwich Research Ethics Committee (Ref. 17.3.5.15). The patients/participants provided their written informed consent to participate in this study.

## Author Contributions

PM and DL: conceptualization, data curation, and funding acquisition. PM, DL, and OR: methodology and review and editing. PM: formal analysis and writing original draft. DL and OR: supervision. All authors contributed to the article and approved the submitted version.

## Funding

Funding has been acquired from the BIAL foundation (Grant number: 359/18), the Society for Psychical Research, the Parapsychology Association, and private funding from Anton Bilton.

## Conflict of Interest

The authors declare that the research was conducted in the absence of any commercial or financial relationships that could be construed as a potential conflict of interest.

## Publisher’s Note

All claims expressed in this article are solely those of the authors and do not necessarily represent those of their affiliated organizations, or those of the publisher, the editors and the reviewers. Any product that may be evaluated in this article, or claim that may be made by its manufacturer, is not guaranteed or endorsed by the publisher.
