# Supplementary material for: An Encounter With the Other: A Thematic and Content Analysis of DMT Experiences From a Naturalistic Field Study
Source: Front Psychol. 2021 Dec 16;12:720717. doi: 10.3389/fpsyg.2021.720717 (PMC8716686; doi:10.3389/fpsyg.2021.720717)
Supplement: Supplementary file 1 [file Data_Sheet_1.DOCX]

**Supplementary Material (SM)**

**1.**

**Encountering Other Beings**

**Interaction & Behaviour: Entities as Interfacing or Independent**

Much of the entities’ interactions with the experiencers have been subsumed by the broader concepts captured by the **Roles**. However, many are best left surviving as modes of *Active involvement* in their own right, such as a couple of psychonauts reporting some phenomenological correlates of *‘*possession’ (2), like RH (Trip 3):

“I knew I was human…but…I’m a creature, experiencing this. In fact…*it* was saying through me, like it was taking over me and saying, ‘Oh it’s very beautiful here isn’t it’… It’s like its able to see through our consciousness, able to experience with us… I’ve become like an alien or something, that I’m using this person’s form in order to experience the world… I didn’t really feel like me, I was and I wasn’t, but mostly I wasn’t, I was something else. And I know [this being]… “I” just wanted to experience the world and it seemed like that was the job, to come into these bodies and experience the world”

*Passive activity* of the beings, as they naturally behaved, most commonly meant encounters unfolding under the gaze of the entities, *‘*watching’ (9) the participants, as referenced by two aforementioned exemplars of curiosity; RH mentions animal-like pod-beings “coming from here and looking down into this universe… And they were looking down, as they were upside down…into me and around me at this universe”. A passive ‘laughing or smiling’ (7) at or with the experiencers followed, naturally aligning with the vastly positive encounters – though sometimes the laughter surrounds the impish energy of the beings, as already elaborated in PD’s trip with his alluring giggling God-child.

Several DMT experiencers were witness to *‘*dancing’ entities (5), for instance MB relates a host of harlequin-esque morph-suits “dancing around…they had all those silks from aerial [gymnastics] ...playing with ribbons.” Her hosts’ “purpose was to make sure every single corner is as pretty as can be”, choregraphing themselves to *‘*beautify’ (2) her DMT environs, as well as apparently doing a spot of *‘*gardening’ (3) “watering different points…and pouring things…with these tiny tubes – they were doing this thing [mimes a tipping hand]”. This was also echoed by JM’s entities, similarly featureless fools hinting at the zodiac symbol of “Aquarius, pouring…at one point as if it were poured over [me]” – as well as BC who mentions another clown-like personage contortedly offering him “a very mini oil barrel”. As with MB’s hurrying harlequins ‘jumping around’ (3), “moving closer to me then moving further away… kept on spinning around”, so too did GC’s (Trip 2) clowns display this frenetic energy, “floating, jumping around… they’re moving very fast…flying over me”.

**2.**

**Exploring Other Worlds**

Typically, this occurs in the first instance via a felt sense of some trajectory of travel (‘Translocation’ subtheme, elaborated in the subsequent report with the near-death experience) – however, eight psychonauts specifically address the idea of breeching some threshold; a *Breaking through the* *veil*, with allusions to piercing the kaleidoscopic membrane only in a minority. Notably, virtually all these experients articulate the breakthrough in terms of a “letting go” during this struggle to facilitate the process. In both GC’s first and second trips he felt and saw himself desperately convulse (purely subjectively), which he interpreted as attempts of his mind to evacuate the body, or surpass the ego:

“And then it was like very strange fight, I was trying to break this reality, to come out of my body and go there… This was my breakthrough basically, my fight to go from this reality to that one… Then the seizures start... I had that kind of seizure maybe, uncontrollable movements… somehow, I’ve the feeling I had a fight with me, my ego, somehow… I think this seizure was in fact my [breakthrough attempt]” (GC, Trip 1)

Finally, again, BB resounds this need to let go before progressing onward, which in turn precedes another liberation after seizing the message of the encounter:

“But I had to let go of something before I could get there… it felt like it was coming there just to pass on a message. Then it was sort of done, it said what it came to say… almost this urgency with which it came. As soon as I was ready, it was just BANG, there… Once I had that very strong feeling – and felt like that was the message it passed on, and it was very clear and obvious – it freed me up again to do what I wanted I guess, within the space, within the feeling”

Most of the experients echoed the sentiment that they underwent an *Emergence into novel reality* *–* inspired by PD’s couching the archetype of this dramatic spiriting away, as he shed off consensus reality, as just “like Dorothy” (from ‘The Wizard of Oz’)*.* While 100% did describe being in a different space of some description, twenty-one made explicit reference to being taken from their normal environs to be “off into another realm of consciousness” (JR) – or as AZ neatly puts it, “it wasn’t what I had seen, but somewhere I had been!” MB, despite admitting her default scepticism, perhaps most clearly makes use of verbal parallels to explain such parallel worlds:

“I was like Oh right! There's this reality! Yeah, right, I've made contact with this reality… I’m not a fan of this theory, but [it was] as if I was tuning in to something different. You have a radio station… It’s as if I was experiencing a completely different reality but I was still here at the same time. Like putting on a VR headset really – [that’s] the best metaphor”

The suddenness of this transition, and the nature of the destination itself, are often described as especially shocking:

“I definitely was not present in this [gestures to room], I was there in that incredible, quite terrifying, but mind-blowing environment… That initial fear is just because it’s so- you’re just taken out, you know I’m sitting here in a house and suddenly you’re not!... you’re in a completely- and it’s all moving, you know you’re transported instantly and it’s all going on around you, it’s like *Zooom, zoom, zoom* and it’s like Fuuuuck!” (FF)

This process, core to the breakthrough DMT experience, had a somewhat different dimension in the case of RH’s first trip, where it was not only he who journeyed to other worlds, but his otherworldly companions bled into this one:

“I knew that only right now is it possible for [the entities] to see into this world… I hear myself saying, ‘Come anytime you want’… They’re looking down into me and around me at this universe...through some sort of thing we’d call a window I guess – about here, up and to the right… This feminine entity [is] giving me the impression that I’m helping to create a gateway, that I’m opening up a gateway for experience… So it doesn’t feel like a one way street, it’s not just me going out there.”

Eleven psychonauts described a *Navigation through space;* consciously negotiating their way through their new-found worlds. The majority were “just flying through these dimensions” (LR), with movement of the essence of ‘floating’ (8). PD loans from his conceptual repertoire to articulate this sense of exploration:

“I was able to move around this splintered situation, and there was definitely…I fucking use drones a lot obviously – imagine a drone following along a pathway with a woman walking with a pram, right? And the drone’s following, so a 3^rd^-person like an out-of-body perspective, I was following this person, *Shwooosh*”

Both MB and MT used resoundingly similar imagery of their floating through a space, somehow mediated by the space itself, which they likened to a “house” or “rooms” – both also incorporating a tunnel-like experience, as MT gave the following when asked if it was related to the “vortices” she mentioned earlier:

“Yeah! It was the…undulation of what was happening that was moving…my consciousness… I had a bit of a choice to go with the current or resist it, but definitely the way the shapes were moving was creating the movement, the pull, and I let my consciousness go into that… you’d follow one energy or hallway or doorway or something and it would *Shhww*, open you up into another space”

**3.**

**Table of all super-ordinate, mid-level and subthemes**

| **An Encounter with the Other** | Clarificatory notes | No. Interviews /36 (%) |
| --- | --- | --- |
| ***Encountering Other Beings*** |  |  |
| *Transformation of persons present* |  | *7 (19)* |
| *DMT personification* | Referring to ‘the DMT’ itself (or its essence) as having agency | *5 (14)* |
| **Sensed Presence** |  |  |
| Presences (No imagery) |  | 6 (17) |
| Omnipresence |  | 5 (14) |
| **Role & Function** |  |  |
| *Helping or nurturing* |  | *19 (53)* |
| The Guide | Assisting the experients in their journey, inviting them in and showing them their world | 8 (14) |
| The Soother | Reassuring | 5 (14) |
| The Playmate | Playing | 5 (14) |
| The Guardian | Protecting | 5 (14) |
| The Healer | Cleansing or repairing, may involve scanning or attempt at resuscitation | 4 (11) |
| The Muse | Inspiring | 1 (3) |
| *Showing or communing* |  | *17 (47)* |
| The Presenter | Offering | 10 (28) |
| The Teacher | Explaining | 9 (25) |
| The Focuser | Directing attention | 5 (14) |
| *Manipulating or controlling* |  | *6 (17)* |
| The Trickster | Comprising the actions and disposition of being mischievous, childish, tempting and laughing at (or joking with) the experient | 3 (8) |
| The Experimenter | Studying the experient, which may involve a clinical scenario, preparing or paralysing them | 2 (6) |
| The Orchestrator | Creating, controlling or holding space | 2 (6) |
| The Consumer | Eating | 1 (3) |
| **Appearance & Features** |  |  |
| Human | Mostly unknown to participant | 6 (17) |
| Other animals | e.g. snakes | 4 (11) |
| *Otherly Creatures – Non-human/Non-animal* |  | *26 (72)* |
| Humanoid |  | 9 (25) |
| Silhouettes / Featureless |  | 8 (22) |
| Clown-like / Jester |  | 4 (11) |
| Octopoid |  | 4 (11) |
| Insectoid |  | 4 (11) |
| Serpentine |  | 3 (8) |
| ‘The Grey- or Mantis’-like | Elongated head/eyes/hands (though re Greys: *without* short height or grey skin of iconic lore) | 2 (6) |
| Therianthropic | Entities have animalistic appearance, which they may shift between | 2 (6) |
| Baby |  | 2 (6) |
| 'Me' or Autoscopy |  | 2 (6) |
| Succubus / Unevolved spirits |  | 2 (6) |
| ‘Navi’-esque | From the movie Avatar | 1 (3) |
| Doctor / Scientist |  | 1 (3) |
| Space invader-like |  | 1 (3) |
| Synapse-like |  | 1 (3) |
| Faerie-like | Though *elf-like* in terms of faerie/animal-like face (as well as trickster-like behaviour), not ‘little’ as per ‘little people’ of DMT lore | 1 (3) |
| Indigenous spirit | Spirit guide of Barquinha cosmology | 1 (3) |
| Disembodied eyes | Comprised of thousands of eyes | 1 (3) |
| Hooded figures |  | 1 (3) |
| Stick creature |  | 1 (3) |
| Bottle-like |  | 1 (3) |
| *Sentient structures* |  | *9 (25)* |
| Sentient geometry |  | 3 (8) |
| Building / Structure (Sentient) |  | 3 (8) |
| Computer symbols/presence |  | 2 (6) |
| Aztec patterns |  | 1 (3) |
| Boxes |  | 1 (3) |
| Candy cane / Pipes |  | 1 (3) |
| *Specific features* |  | *9 (25)* |
| Face(s) *only* |  | 2 (6) |
| Hand(s) *only* |  | 2 (6) |
| Voice *only* |  | 1 (3) |
| Mobius strip |  | 1 (3) |
| Multi-cultural | Various guides of different cultural heritage | 1 (3) |
| Planet-sized |  | 1 (3) |
| Winged |  | 1 (3) |
| Chained |  | 1 (3) |
| Pods |  | 1 (3) |
| *Visual quality* |  | *19 (53)* |
| Self-transforming |  | 8 (22) |
| Geometric |  | 8 (22) |
| Cartoon-like / 2D |  | 6 (17) |
| Colourful |  | 5 (14) |
| Mechanical |  | 3 (8) |
| Hyperdimensional |  | 3 (8) |
| Hideous |  | 1 (3) |
| High-Definition |  | 1 (3) |
| Holographic |  | 1 (3) |
| Metallic |  | 1 (3) |
| Organic-mechanic |  | 1 (3) |
| **Demeanour & Nature** |  |  |
| *Charming and Inviting* |  | *20 (56)* |
| Benevolent / Loving |  | 10 (28) |
| Benign / Friendly |  | 9 (25) |
| Jovial / Happy |  | 4 (11) |
| Motherly |  | 3 (8) |
| Graceful |  | 1 (3) |
| Sensual |  | 1 (3) |
| *Other dispositions* |  | *12 (33)* |
| Curious |  | 4 (11) |
| Childish |  | 3 (8) |
| Urging |  | 2 (6) |
| Panicking |  | 1 (3) |
| Secretive |  | 1 (3) |
| Confused |  | 1 (3) |
| Stubborn |  | 1 (3) |
| Aware of those present |  | 1 (3) |
| Unaware of those present | Entities not cognizant of others in the room such as researchers or participants’ friends | 1 (3) |
| *Mischievous or Jestful* |  | *5 (14)* |
| *Fearsome or Menacing* |  | *3 (8)* |
| *Nature* |  | *17 (47)* |
| One with or of the Beings |  | 10 (28) |
| Familiar |  | 10 (28) |
| Hyper-intelligent |  | 7 (19) |
| Beautiful or Extraordinary |  | 4 (11) |
| Powerful |  | 2 (6) |
| *Expecting subject* |  | *6 (17)* |
| Did not expect |  | 3 (8) |
| Expected |  | 3 (8) |
| *Gender* |  | *17 (47)* |
| Feminine |  | 14 (39) |
| Masculine |  | 7 (19) |
| None |  | 1 (3) |
| **Communication & Messages** |  |  |
| *Communication mode* |  | *14 (39)* |
| Intuition or Telepathy |  | 13 (36) |
| Dance & Gesticulation |  | 3 (8) |
| Visual communication |  | 2 (6) |
| Contactability post-trip |  | 1 (3) |
| Potentially communicative |  | 1 (3) |
| *Messages received* |  | 13 (36) |
| 'The Cosmic Game’ or ‘Cosmic Giggle’ | *The message that the universe is a vast, unified playground for beings to enjoy, and/or possesses an inherent sense of humour* [An extension of *Insight into World* below] | 5 (14) |
| Love for Others and Self | 'Just Love' ; 'Love yourself' ; 'Don’t take people for granted' ; 'Stop getting the fucking hump with him!' ; 'What other people think means fuck all!' | 5 (14) |
| Letting Go | 'Let go…Trust the divine plan' ; 'Surrender, this is a sacred space' ; 'You have to accept your death' | 3 (8) |
| Warning | 'Don’t go there!...You can still turn back!’ ‘They want you to kill someone…They want you to target someone!’ | 2 (6) |
| Insight into the World | 'The answer is right in front of us...Everything already exists' or 'This is just one way of looking at things…It depends where you're looking' | 1 (3) |
| **Interaction & Behaviour** |  |  |
| *Active involvement* |  | *10 (28)* |
| Encompassing or Embracing |  | 2 (6) |
| Opening portals |  | 2 (6) |
| Possessing |  | 2 (6) |
| Preparing |  | 1 (3) |
| Flirting |  | 1 (3) |
| Giving thanks |  | 1 (3) |
| Pulling (subject) away |  | 1 (3) |
| Marching on (subject) |  | 1 (3) |
| *Passive activity* |  | *19 (53)* |
| Watching |  | 9 (25) |
| Laughing or Smiling |  | 7 (19) |
| Dancing |  | 5 (14) |
| Jumping around / Busying |  | 3 (8) |
| Pouring or ‘Gardening’ |  | 3 (8) |
| Hovering |  | 2 (6) |
| Spinning |  | 2 (6) |
| Beautifying / Fixing |  | 2 (6) |
| Wriggling / Crawling |  | 2 (6) |
| Acting |  | 1 (3) |
| Moving on tracks |  | 1 (3) |
|  |  |  |
| ***Exploring Other Worlds*** |  |  |
| *Breaking through the veil* | Subjects explicitly stating there being a barrier of some sort through which they had to pierce to 'breakthrough' | *8 (22)* |
| *Emergence into novel reality* | Subjects explicitly stating the fact they were no longer in ‘this world’ but elsewhere entirely | *21 (58)* |
| *Navigation through space* |  | *11 (31)* |
| Floating / Flying |  | 8 (22) |
| Transporting / Travelling |  | 2 (6) |
| Folded '460 degrees' or Dragged |  | 1 (3) |
| Swallowed |  | 1 (3) |
| **Scene** |  |  |
| *Human worlds* |  | *6 (17)* |
| Street |  | 3 (8) |
| Re-enacted surroundings | Some simulation of the immediate environment and people around the experient | *2 (6)* |
| Building / Structure (Non-sentient) |  | 1 (3) |
| *Natural worlds* |  | *10 (28)* |
| Outer-space | e.g. black holes | 6 (17) |
| Natural landscape |  | 4 (11) |
| Vulva |  | 1 (3) |
| *Artificial worlds* |  | *6 (17)* |
| Mechanism | e.g. 'Conveyor-belts' | 2 (6) |
| Icicle / Waterfall | Digital in nature | 1 (3) |
| Laboratory / Clinical space |  | 1 (3) |
| *Children’s worlds* |  | *3 (8)* |
| Playpen / Nursery |  | 2 (6) |
| Circus-like / Children's book |  | 1 (3) |
| *Nebulous worlds* |  | *13 (36)* |
| Lattice / Gridwork |  | 9 (25) |
| 'Tron-like' or 'Blueprint of Universe' |  | 2 (6) |
| Dark space / Void-like |  | 2 (6) |
| Light space |  | 1 (3) |
| Ether / ‘The fabric’ |  | 1 (3) |
| **Contents** |  |  |
| *Organic objects* |  | *16 (44)* |
| Plants & Flowers |  | 10 (28) |
| Cellular or Subcellular | e.g. DNA (organic) | 4 (11) |
| Molecular or Subatomic |  | 3 (8) |
| 'Intra-Body Experience' | Akin to shamanic practice of viewing the condition of the body from the inside for diagnoses and healing (c.f. OBE) | 1 (3) |
| The Elements |  | 1 (3) |
| *Technological objects* |  | *8 (22)* |
| Mechanics & Devices |  | 4 (11) |
| Satellites & Spacecraft |  | 2 (8) |
| Medicine |  | 2 (8) |
| Car |  | 1 (3) |
| *Infantile objects* | e.g. Push-chairs | *3 (8)* |
| *Geometric objects* |  | *16 (44)* |
| Spherical shapes |  | 7 (19) |
| Hyperdimensional structures | 5/6 being hypercubes | 6 (17) |
| Other polyhedrons |  | 3 (8) |
| Islamic-like sacred geometry |  | 2 (6) |
| Flower of life |  | 1 (3) |
| Mandala |  | 1 (3) |
| Maze |  | 1 (3) |
| *Symbolic objects* |  | *6 (17)* |
| Symbols / Signs | Specific images which had obvious symbolic meaning, which may seem medicinal, religious or ambiguous | 5 (14) |
| Ancient language / Hieroglyphs | Specific images looking like letters, scripts and pictograms of archaic civilisations | 3 (8) |
| Matrix code | Classic strings of numbers and other symbols, ‘Raining code’ as seen in The Matrix trilogy | 2 (6) |
| *Miscellaneous objects* |  | *14 (39)* |
| Serpentine or Cyclic |  | 7 (19) |
| Small barrel / tube |  | 2 (6) |
| Sweets |  | 2 (6) |
| Ladder |  | 1 (3) |
| ‘Magic mirror’ |  | 1 (3) |
| Bible-like book |  | 1 (3) |
| Chalice |  | 1 (3) |
| Antique bathtub |  | 1 (3) |
| ‘Spikey, jangley’ thing |  | 1 (3) |
| **Quality** |  |  |
| *Transforming or Exploding* |  | *7 (19)* |
| *Synthetic textures* |  | *15 (42)* |
| Cartoon-like or Animation |  | 5 (14) |
| Organic-Mechanic |  | 5 (14) |
| High-Definition |  | 4 (11) |
| Holographic or Digital |  | 3 (8) |
| Retro-game or Old |  | 2 (6) |
| *Generic textures* |  | *11 (31)* |
| Very colourful |  | 5 (14) |
| Fractal or Geometric |  | 3 (8) |
| Fluid / Organic |  | 2 (6) |
| Painted |  | 2 (6) |
| Blurry |  | 2 (6) |
| 'Razzmatazz' / Garish |  | 1 (3) |
| Wavey |  | 1 (3) |
| Ornate |  | 1 (3) |
| Jewelled |  | 1 (3) |
| Dream-like |  | 1 (3) |
| ‘Organised Chaos’ |  | 1 (3) |

**4.**

**Graphic of all super-ordinate and mid-level themes**

**
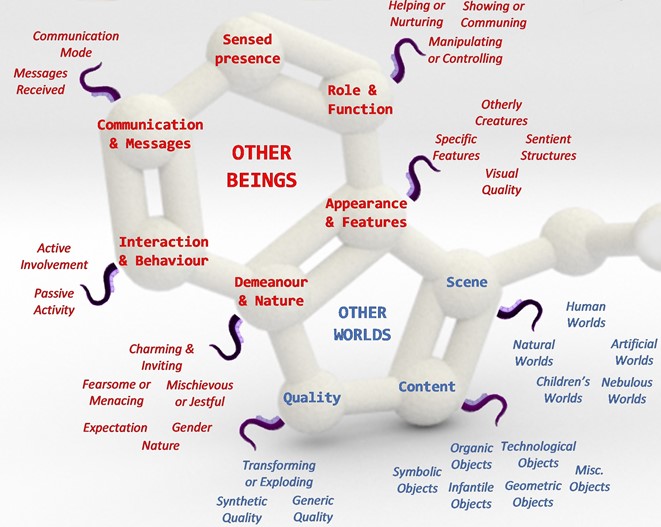
**

**5.**

**Indicative Questions of Semi-Structured Interview (with initial prompts)**

- Please can you describe your DMT experience as best and fully as you can, and as chronologically as you can. Please use all your own words, and try to break down your experience into its detailed elements, while avoiding using terms and concepts from other people or popular culture

- What is the first thing you remember?

- How did your body feel during the experience?

How did your awareness of your body change?

- Did you see any geometric, fractal or entoptic patterns?

Where they colourful, or moving? What was their dimensionality?

- Where did you ‘go’ in your experience?

What did the scene look like?

- Did you have any encounters with other beings in your experience?

What did they look like? What was their approach toward you? Did they communicate with you? Did they interact with you?

- Did you receive any information, or have any particular insights or understanding?

- How was your sense of time affected during the experience?

How long did the experience seem to last? (Versus how long it *did* last)

- How pleasurable was the experience?

What other emotions did you have?

- What was your sense of self like?

In relation to the world around you?

- On a scale from 1-10 where 10 is the most intense and 1 is normal intensity of experience,

how intense did your experience get?
